# Vernal Keratoconjunctivitis: A Systematic Review

**DOI:** 10.1007/s12016-023-08970-4

**Published:** 2023-09-02

**Authors:** Gaia Bruschi, Daniele Giovanni Ghiglioni, Laura Cozzi, Silvia Osnaghi, Francesco Viola, Paola Marchisio

**Affiliations:** 1 Università degli Studi di Milano, Milan, Italy; 2 Fondazione IRCCS Ca’ Granda Ospedale Maggiore Policlinico di Milano, Via della Commenda 9, 20122 Milan, Italy

**Keywords:** Vernal keratoconjunctivitis, VKC, Ocular allergy

## Abstract

Vernal keratoconjunctivitis (VKC) is a chronic, bilateral corneal and conjunctival problem which typically presents in young individuals. VKC is characterized by itching, photophobia, white mucous discharge, lacrimation, foreign body sensation, and pain due to corneal involvement of shield ulcers. Vernal keratoconjunctivitis is categorized within ocular diseases. The diagnosis is clinical, as no sure biomarkers pathognomonic of the disease have yet been identified. The VKC therapy relies on different types of drugs, from antihistamines and topical steroids to cyclosporine or tacrolimus eye drops. In extremely rare cases, there is also the need for surgical treatment for the debridement of ulcers, as well as for advanced glaucoma and cataracts, caused by excessive prolonged use of steroid eye drops. We performed a systematic review of the literature, according to PRISMA guideline recommendations. We searched the PubMed database from January 2016 to June 2023. Search terms were Vernal, Vernal keratoconjunctivitis, and VKC. We initially identified 211 articles. After the screening process, 168 studies were eligible according to our criteria and were included in the review. In this study, we performed a systematic literature review to provide a comprehensive overview of currently available diagnostic methods, management of VKC, and its treatments.

## Introduction

Vernal keratoconjunctivitis (VKC) is a chronic bilateral keratoconjunctivitis typical of children. It usually manifests in the first decade of life [1], although some cases are described also in adults [2].

Its prevalence shows extreme geographic variability (Table 1). The highest incidence is reported in African countries, with incidence decreasing in direct proportion to the distance from the equator.

**Table 1:** VKC epidemiology

Author	Year	Country	Prevalence
Alemayehu et al. [3]	2019	Ethiopia	5.2–7.3%
Marey et al. [4]	2017	Egypt	3.3%
Smedt et al. [5]	2011	Rwanda	4.0% between 7 and 14 years
Bremond-Gignac et al. [6]	2008	Italy	0.27%
Bremond-Gignac et al. [6]	2008	Finland	0.007–0.084%
Bremond-Gignac et al. [6]	2008	Sweden	0.012–0.087%
Bremond-Gignac et al. [6]	2008	Norway	0.003–0.019%
Bremond-Gignac et al. [6]	2008	France	0.007–0.033%
Bremond-Gignac et al. [6]	2008	The Netherlands	0.006–0.046%

VKC is characterized by itching, photophobia, white mucous discharge, lacrimation, foreign body sensation, and pain due to corneal involvement of shield ulcers. The pathognomonic signs of VKC are Trantas dots (aggregations of epithelial cells and eosinophils), cobblestone giant papillae at the upper tarsal lids, and shield ulcers [7]. Other signs described are conjunctival hyperemia, gelatinous infiltrate at the limbus, neovascularization of the cornea, and pseudogerontoxon [8]. There are three forms of VKC: tarsal, limbal, and mixed. The tarsal form is characterized by papillae in the upper tarsal lid, while the limbal form by gelatinous infiltrates in the limbus (characterized by an infiltration of lymphocytes, plasma cells, macrophages, basophils, many eosinophils, and conjunctival goblet cells [9]), Trantas dots (white nodules composed of eosinophils and epithelial debris located at the limbus [9]), and, eventually, punctate keratitis and shield ulcers [1]. In the mixed form, both the cornea and the tarsal conjunctiva are involved.

Although VKC usually resolves after puberty, it can lead to severe visual impairments if the therapy is not adequate. The patient could develop progressively visual loss (reported in 5–30% of cases), shield ulcers, cataracts, and glaucoma, caused by excessive prolonged use of steroid eye drops [1].

VKC therapy relies on different types of drugs. The mild form is usually treated with antihistamine eye drops, mast cell stabilizers, eosinophil inhibition drops (e.g., ketotifen), and short cycles of topical steroids. Moderate and severe forms usually require instead a prolonged course of steroids to control signs and symptoms of the disease, and/or an immunomodulatory therapy with cyclosporine or tacrolimus eye drops [7]. In extremely rare cases, there is also the need for surgical treatment for the debridement of ulcers, as well as for advanced glaucoma and cataracts [1].

VKC is classified among ocular allergies, representing one of the 6 subtypes of ocular allergy (along with seasonal allergic conjunctivitis (SAC), perennial allergic conjunctivitis (PAC), atopic keratoconjunctivitis (AKC), contact blepharoconjunctivitis (CBC), and giant papillary conjunctivitis (GPC)). However, the underlying causes of VKC remain unclear. The pathogenesis likely involves a variable combination of genetic and endocrinological pathways, as well as immune-mediated and environmental factors [1].

In this study, we performed a systematic review of the literature to provide a comprehensive overview of the currently available diagnostic methods for VKC, its management, and its treatments.

## Materials and Methods

We performed a systematic review of the literature, according to the Preferred Reporting Items for Systematic Reviews and Meta-analyses (PRISMA) guideline recommendations [10]. We searched the PubMed database from January 2016 to June 2023. We did not restrict the research to language. Search terms were Vernal, Vernal keratoconjunctivitis, and VKC.

In this review, we included systematic and narrative reviews, clinical trials, retrospective and prospective observational studies, case series, and case reports. All the studies were subsequently divided into two categories: those discussing VKC diagnosis and those discussing VKC therapy.

We also included in this review studies describing VKC manifestations and treatment in adult patients.

### Study Eligibility and Quality Assessment

We included in this review all articles that provide diagnostic or therapeutic data on VKC. At first, we screened titles and abstracts to discover eligible studies, and then, we analyzed all full texts for the final evaluation.

The inclusion criteria we used to determine if an article was appropriate were (1) VKC populations (both children and adults), (2) diagnosis of VKC made with specified diagnostic criteria, and (3) report of epidemiological, clinical, diagnostic, and/or therapeutic data.

Exclusion criteria were as follows: (1) not the relevant topic (not appropriate population or not appropriate outcome), (2) non-original studies (e.g., duplicate articles or comments), and (3) in vitro studies.

The quality of the eligible studies was evaluated using different methods according to the study design: the Amstar 2 Checklist for Systematic Reviews [11], the SANRA scale for Narrative Reviews [12], the Jadad score for Randomized Clinical Trials (RCT) [13], the Strobe Checklist for the Observational Studies [14], the Joanna Briggs Institute (JBI) Critical Appraisal Checklist for Case Reports [15], and the Joanna Briggs Institute (JBI) Critical Appraisal Checklist for Case Series [15].

From each study, we considered information regarding study design, date of publication, country of origin, setting, characteristics of the population sample, objective of the study, and outcome measure.

## Results

**Fig. 1:**
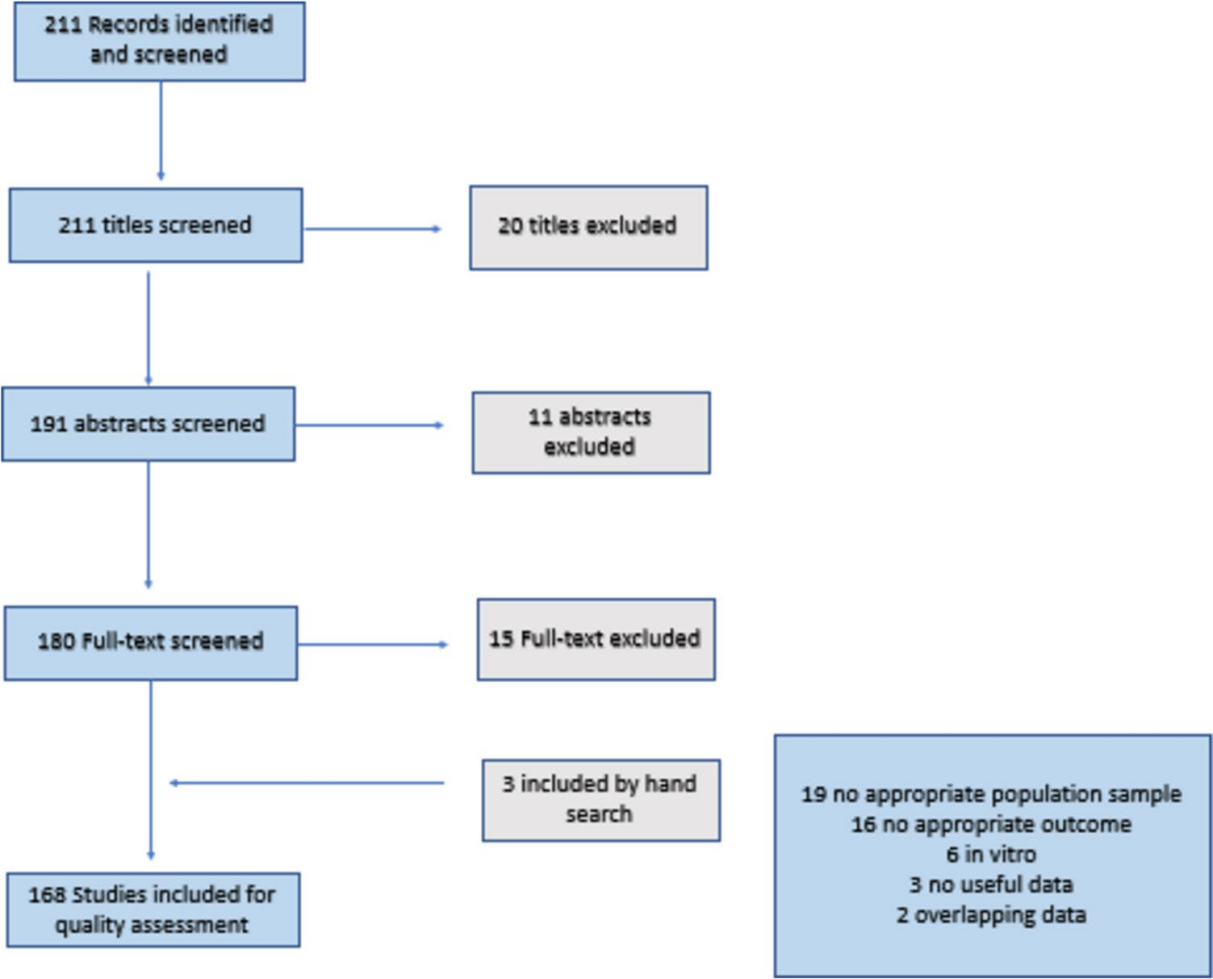
Flow chart of the study selection

The selection process is shown in Fig. 1.

We initially identified 211 articles. Twenty studies were excluded from the title, 11 studies were excluded after reading the abstracts, and 15 studies were excluded after the full-text analyses. Nineteen studies were excluded for an inappropriate population (only AKC, SAC, or PAC patients), 16 studies for an inappropriate outcome, 6 studies for having been conducted only in vitro, 3 studies for providing non-useful data (studies still in progress or future study protocols not yet implemented), and 2 studies for providing overlapping data using the same study population as a previously included study.

After the screening process, 168 studies were eligible according to our criteria and were included in the review.

**Fig. 2:**
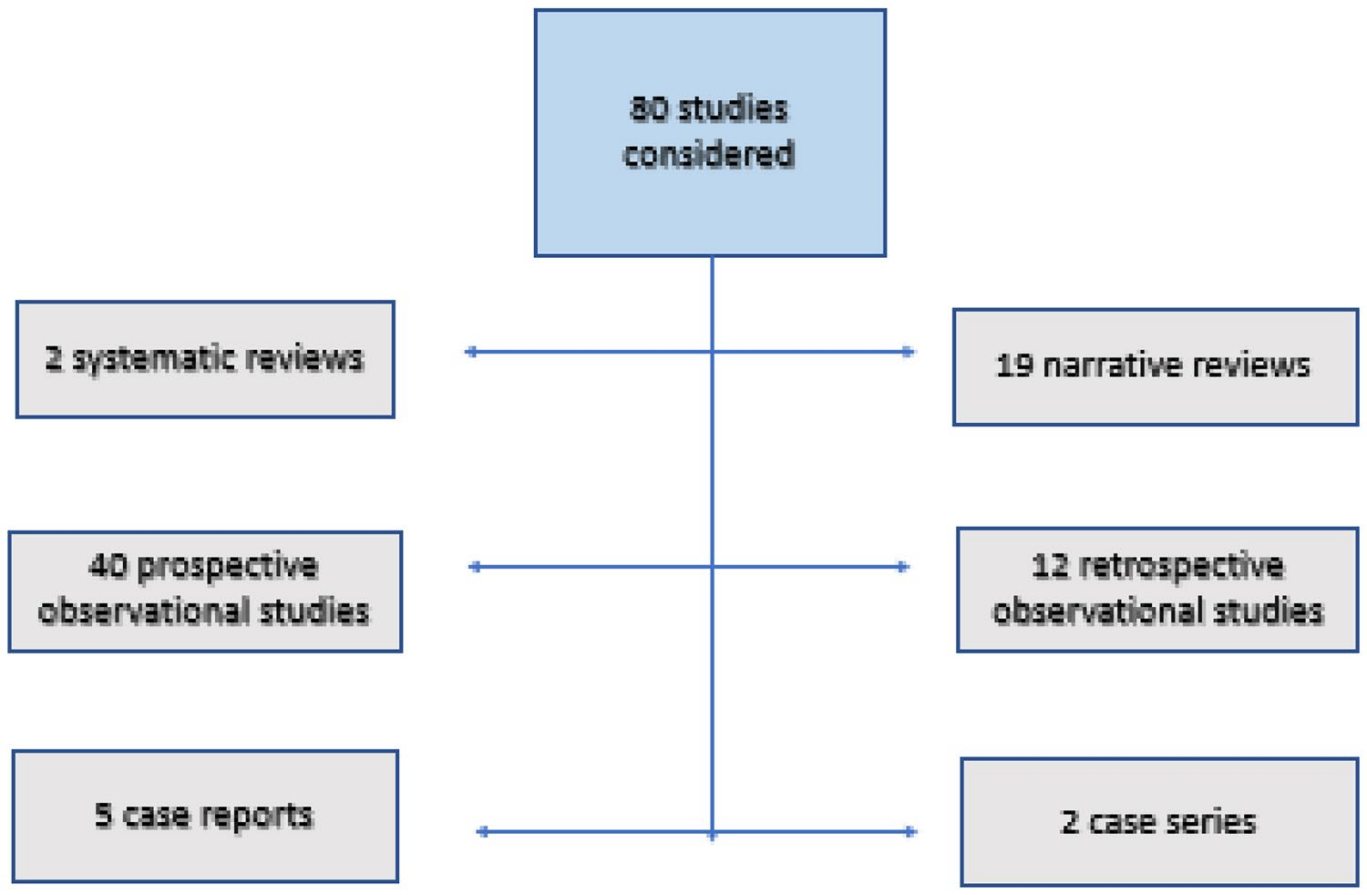
Flow chart of VKC diagnosis studies

**Fig. 3:**
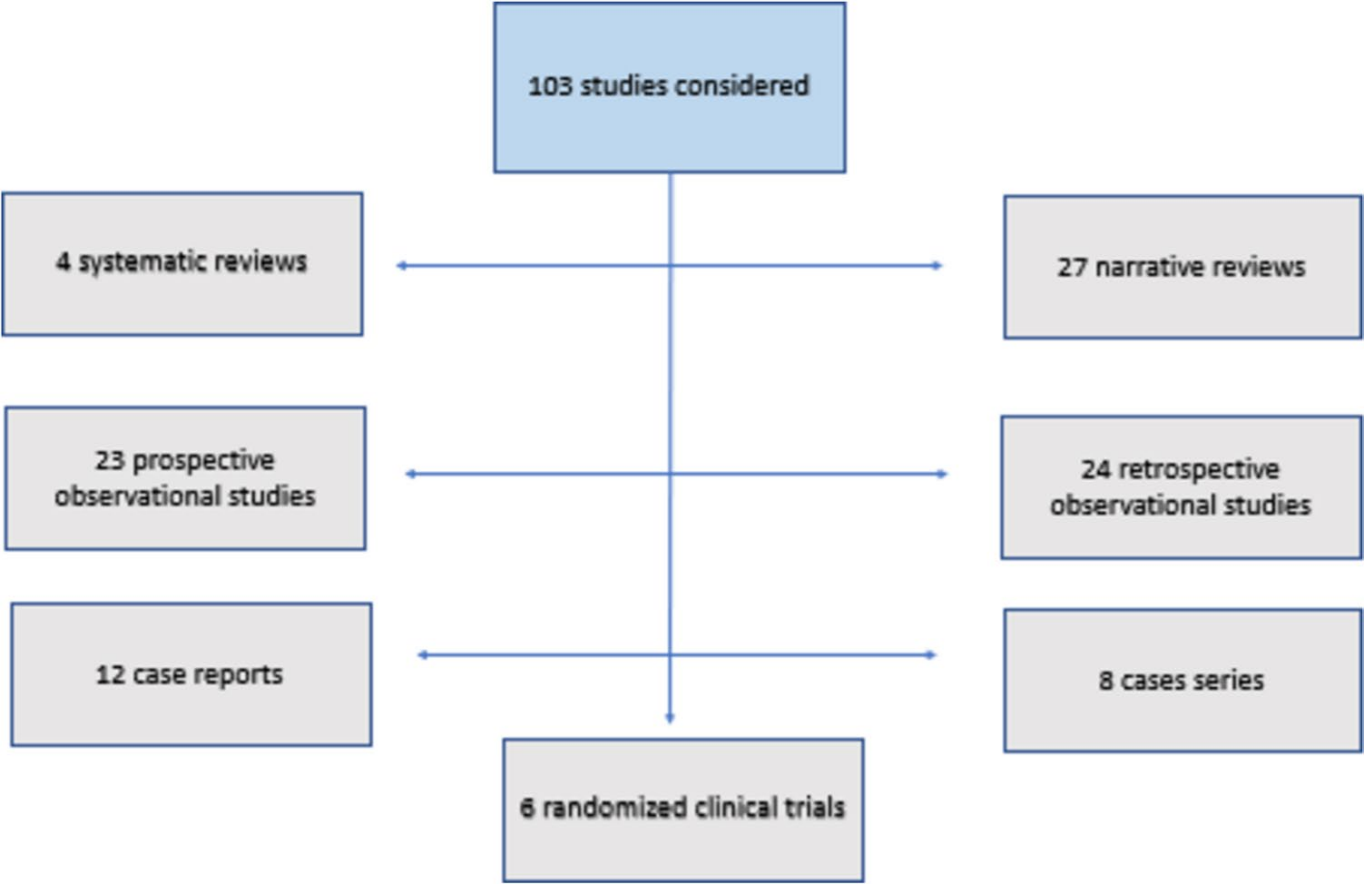
Flow chart of VKC treatment studies

Among the 168 studies finally considered, 65 concerned VKC diagnosis, 88 studies described VKC therapies, and 15 studies discussed both diagnosis and therapy. The flow chart of the final studies considered for diagnosis and treatment is represented in Figs. 2 and 3. Two of the studies included in the treatment were considered both as a narrative review and as a case series [16, 17]. Three articles were included after hand research [18–20].

Characteristics of the included studies are reported in Tables 2, 3, 4, 5, 6, 7, 8, 9, 10, 11, 12, 13, and 14.

**Table 2:** Diagnosis systematic review

Author	Year	Country	N VKC studies	No. of VKC patients	Median age	Outcome	Results	Amstar score
Leonardi et al. [21]	2020	Italy	13	N/A	N/A	To identify efficacy endpoints being proposed in clinical trials for VKC	Penalty-adjusted corneal staining score at month X is calculated as follows: corneal fluorescein staining (CFS) (baseline) − CFS (month X) + penalty/ies (+ 1 penalty for rescue medication, and + 1 penalty for corneal ulceration). This score is a reliable method to assess corneal modification during time and to evaluate the efficacy of novel drugs	Low
Rasmussen et al. [22]	2022	Denmark	29	2122	N/A	To determine the prevalence of allergic sensitization in patients with VKC	The prevalence of allergic sensitization in patients with VKC is 57.7% and mostly toward inhaled allergens. The most frequent positive allergens are house dust mites and pollen	High

*N/A* non-available information

**Table 3:** Diagnosis narrative reviews

Author	Year	Country	N VKC studies	No. of VKC patients	Median age	Outcome	Results	SANRA scale
Gokhale [23]	2016	India	N/A	N/A	N/A	To evaluate VKC severity grading system	Based on signs and symptoms, VKC is classified as tarsal, limbal, or mixed forms (72%) and into mild, moderate-intermittent, moderate-chronic, severe, and blinding disease. 36% had a perennial form of VKC. 6% of patients developed cataracts and 4% glaucoma. In 12% of cases, the disease was still present over 20 years of age	Low
Kraus [18]	2016	USA	N/A	N/A	N/A	To describe VKC manifestations	VKC is characterized by itching, tearing, mucous discharge, and photophobia. The main signs are giant papillae and Trantas dots. The disease usually lasts 4 to 10 years and resolves after puberty	Critically low
Berger et al. [19]	2017	USA	N/A	N/A	N/A	To present an algorithm for ocular allergy diagnosis	An ad hoc algorithm based on a comprehensive medical history and physical examination could allow the differential diagnosis between the various forms of allergic conjunctivitis	Medium
Takamura et al. [24]	2017	Japan	N/A	N/A	N/A	To propose a guideline for ocular allergy	Characteristic VKC lesions are exfoliated superficial punctate keratitis, shield ulcer, and corneal plaque. In serum and lacrimal fluid, total IgE antibodies and eosinophils are increased	Critically low
Thong [25]	2017	Singapore	4	1800	15.7	To describe ocular conjunctivitis in Asia	VKC is characterized by giant papillae, fibrinous discharge, Trantas dots, lower eyelid creasing (Dennie’s lines), and pseudo membrane on the upper lid	Low
Sacchetti et al. [26]	2018	Italy	N/A	N/A	N/A	To review VKC pathogenesis	VKC diagnosis is based on the clinical history and eye evaluation and can be confirmed through allergology tests	Medium
Fauquert [27]	2019	France	N/A	N/A	N/A	To present the salient points concerning VKC diagnosis	The child with photophobia, secretions, eye pain, and visual impairment should refer to an ophthalmologist to confirm diagnosis, rule out differential diagnoses, and consider the possibility of local corticosteroid therapy	Medium
Bielory et al. [28]	2020	USA	N/A	N/A	N/A	To describe ocular allergy classification, its main signs, and symptoms	Ocular allergies are characterized by itching, redness, and dry eye. Skin prick test, patch test, conjunctival provocation test, tear film evaluation, and corneal staining could help perform the differential diagnosis between the various form of ocular allergies	Low
Di Zazzo et al. [2]	2020	Italy	N/A	N/A	N/A	To describe VKC in adults	Adult variants of VKC have the same clinical manifestations as the classic form but show higher inflammatory response and increased risk of chronic fibrotic sequelae	Low
Shoji [29]	2020	Japan	N/A	N/A	N/A	To describe possible biomarkers of ocular disease	In patients with VKC, tear eosinophil cationic protein (ECP) and eotaxin-2 levels correlate with disease severity (*p* < 0.01). Other potential biomarkers of VKC should be osteopontin and periostin levels	Medium
Brindisi et al. [30]	2021	Italy	N/A	N/A	N/A	To provide an overview of VKC	VKC symptoms are well known, but they can overlap and be mistaken with allergic conjunctivitis. Diagnostic criteria and severity grading are not standardized yet	Low
Ghiglioni et al. [31]	2021	Italy	N/A	N/A	N/A	To describe VKC manifestations	VKC, as AKC, represents a disease of particular complexity and potential severity in its management among the various allergic eye diseases	Medium
Sacchetti et al. [32]	2021	Italy	N/A	N/A	N/A	To review VKC pathogenesis	Increasing understanding of the pathogenic mechanisms behind VKC may lead to the identification of novel biomarkers for diagnosis and/or potential therapeutic targets to improve the management of this challenging condition	Medium
Singh et al. [33]	2021	India	N/A	N/A	N/A	To provide an overview of barrier dysfunction in ocular allergy	Whether barrier dysfunction precedes and predisposes to ocular allergy development is still not clearly understood; however, it maintains and contributes to the vicious cycle of allergic inflammation by facilitating paracellular transport of allergens, pathogens, pollutants, and other harmful triggers	Medium
Wajnsztajn and Solomon [34]	2021	Israel	N/A	N/A	N/A	To review the association between VKC and keratoconus	Keratoconus prevalence can be as high as 26.8% among VKC patients, whereas abnormal corneal topography may appear in up to 71% of them. It is more severe and progresses faster in the setting of VKC (*p* < 0.05), with remarkable visual deterioration and with an increased need for keratoplasty	Medium
Kaur and Gurnani [35]	2022	India	N/A	N/A	N/A	To describe the etiology of VKC, its differential diagnosis, and complications	The diagnosis of VKC is based on clinical examination. The typical history and clinical signs lead to a straightforward diagnosis in most cases. Different staining techniques and associated scoring systems have been described to grade the severity of VKC	Low
Mehta et al. [36]	2022	Singapore	N/A	N/A	N/A	To develop recommendations for the assessment and diagnosis of VKC in Asia	The diagnosis of VKC relies on the presence of tarsal papillae and Trantas dots in the limbus. Across Asia, the most widely used model for assessing disease’s severity is the Bonini scale	High
Leonardi et al. [37]	2023	Italy	N/A	N/A	N/A	To highlight different clinical features and diagnostic criteria for VKC and AKC	Specific diagnostic criteria should guide an early diagnosis and prognosis also in relation to specific treatment needs. Even though VKC/AKC overlaps exist, a child with atopic dermatitis, facial and eyelid involvement should be considered AKC patients with the prognosis of having the disease in the adulthood	Medium
Nche et al. [38]	2023	Cameroon	N/A	N/A	N/A	To review current literature from sub-Saharan Africa	There is a variable prevalence of VKC in sub-Saharan area, up to 32.9% of children, especially in male individuals less than 5 years old. The mixed form of VKC is the most frequent form seen, and conjunctival pigmentation might be an early diagnostic sign	Low

*N/A* non-available information

**Table 4:** Diagnosis prospective observational studies

Author	Year	Country	No. of VKC patients	Median age	Outcome	Results	Strobe checklist
Fujishima et al. [39]	2016	Japan	7	24.6	To assess the role of periostin as a biomarker for allergic conjunctivitis	Tears from patients with ocular allergic disease showed significantly higher periostin levels than allergic patients without conjunctivitis (*p* < 0.05), with maximal levels in AKC and VKC (*p* < 0.001)	Medium–low
Leonardi et al. [40]	2016	Italy	9	10.4	To evaluate Heat Shock Proteins (Hsp) chaperone expression in the conjunctiva of VKC patients	Hsp27, Hsp40, Hsp70, and Hsp90 levels were higher in patients’ conjunctiva than in controls (*p* < 0.05). Their evaluation may become useful in VKC diagnosis	Medium–low
Inada et al. [41]	2017	Japan	19	23.4	To investigate the expression of histamine H1 and H4 receptors mRNA (H1R and H4R) on the ocular surface	H1R and H4R expressions were higher in the active than in the stable stage subgroup of AKC/VKC patients (*p* < 0.05), without significant differences between the AKC and VKC groups	High
Shoji et al. [42]	2017	Japan	14	16.8	To evaluate thymus activation-regulated chemokine (TARC), eotaxin-2, and IL-16 tear levels in allergic conjunctival disorders (ACDs)	Tear levels of CCL17/TARC, CCL24/eotaxin-2, and IL-16 in VKC and AKC patients were significantly higher than in patients with AC (*p* < 0.01)	Medium–low
Zicari et al. [43]	2017	Italy	47	8.6	To evaluate serum vitamin D in children affected by VKC and the relationship between its levels and disease severity	Children affected by VKC had lower levels of vitamin D compared to healthy controls (*p* < 0.0001). After 6 months of cyclosporine therapy, these levels increased (*p* = 0.004) but were lower than in healthy controls (*p* < 0.05)	Medium
Costa Andrade et al. [44]	2018	Brazil	29	13.3	To investigate the expression of galectin-3 in the conjunctiva of VKC patients	Patients with VKC exhibited increased levels of Gal-3 in the conjunctiva compared with control (*p* < 0.001). Gal-3 could be used as a biomarker of VKC	Medium–low
Nebbioso et al. [45]	2018	Italy	21	8.6	To evaluate the concentration of the vascular endothelial growth factor (VEGF) in tear and blood samples from patients with VKC	VKC patients showed higher levels of VEGF in tears than healthy controls (*p* < 0.05), but no difference of VEGF levels in the blood (*p* = 0.29)	Medium–low
Nebbioso et al. [46]	2018	Italy	47	8.8	To evaluate lacrimal film, tear ferning test (TFT) modifications, and density of conjunctival goblet cells in VKC patients	VKC patients had an increase in TFT at baseline than healthy subjects (*p* < 0.001). After cyclosporine treatment, there was an improvement in TFT score *p* = 0.044) and density of conjunctival goblet cells (*p* = 0.044)	Medium–low
Bruschi et al. [47]	2019	Italy	56	9.4	To assess the most common cell types present in the conjunctiva of children with VKC	Epithelial cells and mast cells were more prevalent in the conjunctiva of not-treated patients (*p* = 0.01). Steroidal eye drops decreased the number of neutrophils and eosinophils (*p* = 0019 and *p* = 0.055)	Medium–low
Leonardi et al. [48]	2020	Italy	25	12.6	To identify differences in gene expression between VKC and normal subjects and to evaluate the expression of pattern recognition receptors (PRRs)	The increased expression of several chemotactic factors and co-stimulatory signals required for T cell activation confirms that VKC is mostly cell-mediated with local eosinophilia. The multiple expression of PRRs suggests a role of host–pathogen interaction in VKC development	Medium–high
Zicari et al. [49]	2020	Italy	24	9.6	To assess the oxidative stress in VKC patients and controls and to study the effect of cyclosporine A (CsA) on oxidative stress in these patients	VKC untreated children had significantly higher values of hydrogen peroxide (H_2_O_2_) in serum and tears with respect to VKC treated children	High
Ahmed et al. [50]	2021	Egypt	80	10.0	To determine the prevalence of keratoconus (KC) among children with ocular allergy	The overall prevalence of KC was 34%. Risk factors for the development of KC in patients with ocular allergy were age, duration of symptoms, systemic atopy, and VKC	High
Çağlayan et al. [51]	2021	Turkey	72	N/A	To compare the corneal and lens densitometry values between VKC patients and healthy individuals	An increase in the lens densitometry values was observed in patients with moderate and severe VKC compared to healthy individuals	Medium
Horinaka et al. [52]	2021	Japan	19	25.2	To evaluate the presence of ocular surface mucin in patients with AKC and VKC	Ocular surface mucin in patients with AKC/VKC is altered following the clinical severity of the disease	High
Kavitha et al. [53]	2021	India	55	10.4	To assess the posterior corneal elevation (PCE) in children with VKC compared to controls	Children with VKC have significantly higher PCE (*p* < 0.001). All VKC children should be screened for the development of keratoconus	High
Mashimo et al. [54]	2021	Japan	7	N/A	To investigate the role of oncostatin M (OSM) in the pathogenesis of VKC	OSM concentration was higher in the tear fluid of VKC patients than in that of the healthy controls, with strong expression of OSM mRNA in the giant papillae	Medium
Menta et al. [55]	2021	India	87	12.5	To explore if sphingolipid metabolism on the ocular surface has a contributory role in the refractoriness of VKC	Altered sphingolipid metabolism in the ocular surface results in low tear ceramide and sphingosine levels in severe/very severe VKC compared with the mild/moderate cases	High
Messina et al. [56]	2021	Italy	23	15.0	To identify specific changes of N-glycome in tears and to recognize possible glyco-biomarkers in AKC and VKC	VKC and AKC patients and controls show three distinct patterns in terms of relative intensities for some N-glycan structures	Medium
Muamba Nkashama et al. [57]	2021	Congo	400	N/A	To describe the clinical characteristics of VKC in Kinshasa, to evaluate the sensitization profile and associated factors	34.5% of children had a positive skin prick test to at least one allergen, in particular for *Dermatophagoides pteronyssinus*, *Blomia tropicalis*, and cockroach	Medium
Sacchetti et al. [58]	2021	Italy	18	10.7	To evaluate changes of tear soluble sCD14 and conjunctival CD14, TLR-4 and 9 expressions in patients with VKC in the active and quiescent phases	Tear sCD14 and conjunctival CD14, TLR4, and TLR-9 decreased during the active phase. They may represent biomarkers of VKC activity and novel therapeutic targets	Medium
Sorkhabi et al. [59]	2021	Iran	39	18.3	To evaluate the serum vitamin D levels of patients with VKC	Patients affected by VKC had statistically significant lower 25(OH)D levels (27.64 ± 8.50 ng/mL) than healthy subject group (35.96 ± 11.34 ng/mL) (*p* = 0.001)	Medium
Vishwakarma et al. [60]	2021	India	30	9.2	To compare ocular surface microbiome and its antibiotic sensitivity in VKC with normal ocular surface	*Staphylococcus* species were identified in 70% VKC group and 57% control group. Fluoroquinolone resistance was more prevalent among higher grades of VKC (50%) (*p* < 0.01) and was observed in 46% of VKC patients and 23% of the control group (*p* < 0.01)	High
Yılmaz et al. [61]	2021	Turkey	51	17.5	To evaluate corneal and crystalline lens densitometry in patients with VKC	Posterior corneal astigmatism is increased in VKC cases in comparison with age- and gender-matched controls. Lens clarity is likewise decreased in VKC cases with respect to controls	High
Zhang et al. [62]	2021	China	23	9.6	To evaluate the association between AC and health-related quality of life (HRQoL) in children and their parents	AC has a negative association with HRQoL for children and their parents, especially in children with VKC/AKC or higher corneal fluorescein staining scores	High
Kausar et al. [63]	2022	Pakistan	109	22.7	To evaluate the epidemiological aspects of allergic conjunctivitis in Pakistan	VKC is the most common type of allergic conjunctivitis (46.2%), prevalent in males of age < 20 years. Allergic rhinitis was the most common co-morbidity, followed by dermatitis	High
Micera et al. [64]	2022	Italy	22	17.8	To describe local tissue remodeling in a cohort of adult VKC patients	Increased local conjunctival androgen receptors were detected in patients with adult variants compared to classic childhood VKC and healthy subjects	High
Ninomiya et al. [65]	2022	Japan	4	N/A	To investigate the role of oncostatin M (OSM) in VKC	OSM has important roles in severe, prolonged allergic inflammation by inducing epithelial barrier dysfunction and IL-33 production by conjunctival fibroblasts	Medium
Sabu et al. [66]	2022	India	68	9.8	To compare the ocular surface parameters of children with VKC with healthy controls	Severity of VKC was found to be positively correlated with grade of squamous metaplasia (*p* < 0.001) and negatively correlated with noninvasive tear film break-up time and lipid layer thickness	High
Sacchetti et al. [67]	2022	Italy	18	10.7	To evaluate changes of sCD14, TLR-4, and 9 expressions in patients with VKC in the active and quiescent phases	Expression of tear sCD14 and of conjunctival CD14, TLR-4, and TLR-9 was significantly decreased during active inflammation	Medium
Singh et al. [68]	2022	India	135	8.3	To study the demographic and clinical characteristics of childhood and adult onset VKC during COVID-19 pandemic	Adult onset VKC included 10.4% of the total patients. The disease was more common in males, with a male to female ratio of 2.5:1. Limbal VKC was the most common presentation found in 61.5% of the patients	High
Syed et al. [69]	2022	Malaysia	4	9.7	To identify the miRNA expression profile in the tears of children with VKC vs controls	A total of 51 miRNAs were differentially expressed in the tears of children with VKC. Of the 51 miRNAs, 48 were significantly upregulated, while 3 miRNAs were significantly downregulated	High
Albadawi et al. [70]	2023	Egypt	71	8.1	To evaluate the corneal epithelial thickness by anterior segment OCT in children with VKC	Corneal epithelial thickness mapping showed significant superior thinning (51.07 ± 4.11) μm in VKC group compared to healthy controls (52.54 ± 2.01) μm (*p* = 0.008)	High
Csorba et al. [71]	2023	Hungary	20	N/A	To investigate the morphological characteristics of corneal microstructure in the quiescent phase of VKC	Langerhans cell density, morphology, and field area were significantly higher in the VKC group than in healthy controls (*p* = 0.005, *p* < 0.001, and *p* < 0.001, respectively)	High
Dubbaka et al. [72]	2023	India	152	11.4	To evaluate presence of perilimbal pigmentation (PLP) in Indian patients with VKC	PLP was present in 81 cases (53.29%; *p* < 0.001), of which 15 cases (18.5%) had this pigmentation in all the four quadrants	High
Gupta et al. [73]	2023	India	87	9.1	To evaluate dry eyes in children with VKC	Dry eyes are seen in two-thirds of pediatric VKC. Evaluation of dry eyes should be incorporated in their clinical evaluation	High
Ito et al. [74]	2023	Japan	26	11.4	To investigate the concentration and source of galectin-3 in the tears of patients with VKC	High concentrations of galectin-3 were detected in the tears of patients with VKC. The concentration showed significant correlation with the severity of corneal epithelial damage	Medium
Mazumdar et al. [75]	2023	India	30	N/A	To assess the prevalence of dry eye in different subsets of allergic conjunctivitis (AC)	This study revealed a high prevalence of dry eye disease (DED) in patients with AC. Among these, perennial AC had the highest percentage of DED, followed by SAC and VKC	High
Thiagarajan et al. [76]	2023	Malaysia	43	N/A	To evaluate the corneal topographical changes in VKC subjects using OCULUS Pentacam	Central corneal curvature and astigmatism were significantly higher in VKC subjects compared to the normal population (*p* < 0.05). The minimal pachymetry was significantly lower with a longer duration of VKC (*p* < 0.05)	High
Yilmaz et al. [77]	2023	Turkey	80	13.1	To investigate changes in topometric corneal indices and proclivity toward corneal ectasia, keratometric indices, and anterior chamber dimensions in palpebral VKC	Significantly higher mean topometric indices in VKC (*p* = 0.001) indicate a proclivity for corneal ectasia, which could be attributed to general changes in the corneal ultrastructure caused by persistent itching-induced eye rubbing	High
Zhang et al. [78]	2023	China	20	11.0	To explore the corneal biomechanical properties (CBPs) of patients with VKC	The corneas of VKC patients were softer and more protruded compared with the control group. VKC patients in limbal form were more inclined to develop keratoconus	High

*N/A* non-available information

**Table 5:** Diagnosis retrospective observational studies

Author	Year	Country	No. of VKC patients	Median age	Outcome	Results	Strobe checklist
Gupta et al. [79]	2015	India	51	12.0	To determine prevalence, risk factors, and severity of visual loss in steroid-induced glaucoma (SIG)	Of the 1259 children with glaucoma studied, 51 had received topical steroids for VKC. Of these, 45% required filtering surgery	Medium–low
Gómez-Henao et al. [80]	2018	Spain	32	12.1	To evaluate clinical manifestation and quality of life of VKC patients	VKC commonest symptoms were pruritus (75%), photophobia (50%), and red eye (43.8%). VKC patients had low quality of life scores	High
Ghiglioni et al. [81]	2019	Italy	71	9.6	To evaluate the relationship within ocular symptoms control and serum level of vitamin D in VKC	Vitamin D levels were higher when measured after preceding local therapy, especially in VKC limbal form (*p* = 0.02) and in phototypes II and III (*p* = 0.02)	High
Senthil et al. [82]	2019	India	4062	12.0	To describe the clinical features and outcome of steroid-induced glaucoma (SIG) in VKC	The prevalence of SIG was 2.24%. IOP was medically controlled in 66%, and 34% required surgical treatment	Medium
Jongvanitpak et al. [83]	2020	Thailand	20	7.9	To describe clinical manifestation of ocular allergy	VKC was characterized by papillae and Trantas dots. Giant papillae and corneal ulcers were pathognomonic of VKC	Medium
Artesani et al. [84]	2021	Italy	70	8.1	To evaluate health-related quality of life (HRQoL) in children at VKC diagnosis	The QUICK questionnaire could be a useful tool to evaluate HRQoL in children with VKC	High
Donthineni et al. [85]	2021	India	7	22.1	To describe the histopathological characteristics of limbal stem cell deficiency (LSCD) due to VKC	The histopathological features of LSCD in VKC reveal some distinctive characteristics, like the presence of epithelial down growth, eosinophilic infiltration, and epithelial solid and cystic implants	High
Ghauri et al. [86]	2021	UK	9	6.0	To understand the impact of VKC on daily life	Families of children with VKC experience delays in receiving an accurate diagnosis and a lack of information and emotional support	Medium
Wadhwani et al. [87]	2021	India	65	N/A	To determine the knowledge and attitude about VKC in caregivers	A total of 69.2% of caregivers were not aware of the symptoms of the disease, and 83% of caregivers were unaware of the side effects of eye drops used	Medium
Artesani et al. [88]	2022	Italy	29	8.7	To estimate the impact of reduced sunlight exposure in patients with VKC during the imposed lockdown period for COVID-19	No significant changes in signs and symptoms were observed comparing 2020 to 2019 values. Ten (34.4%) patients did benefit from the reduced sunlight exposure. The increased use of bright screens was associated with worsening of VKC severity	High
Masini et al. [89]	2022	Italy	161	10.9	To evaluate the impact of screen exposure on children with VKC during the COVID-19 lockdown	Mean scores of signs and symptoms increased homogeneously when studying patients exposed to longer screen time. There was not a significant reduction in clinical manifestations between 2019 and 2020	High
Yang et al. [90]	2023	China	40	12.3	To investigate the clinical features of VKC in Tibet	Typical limbal and subconjunctival lesions, such as Horner–Trantas dots, occurred in 87.5% of the patients. In some patients, the nodules infiltrated and even covered the cornea, leading to blindness	Medium

*N/A* non-available information

**Table 6:** Diagnosis case series

Author	Year	Country	N VKC patients	Median age	Outcome	Results	JBI checklist
Soleimani et al. [91]	2016	Iran	2	39.5	To report 2 cases of Splendore-Hoeppli phenomenon, a late clinical finding in VKC	Splendore-Hoeppli phenomenon (granulomatous inflammation with deposition of eosinophilic matter in the conjunctiva) was observed as a late manifestation in adult patients suffering from VKC in infancy	Low
Jaffet et al. [92]	2021	India	3	19.0	To report the clinical outcomes and histopathological and immunohistochemistry (IHC) features in VKC	The expression of transient progenitor cells in the scarred corneas of VKC patients suggests that the limbal stem cell dysfunction is likely partial and self-renewal of limbal stem cells is plausible	Medium -low

*N/A* non-available information

**Table 7:** Diagnosis case report

Author	Year	Country	No. of VKC patients	Age	Outcome	Results	JBI checklist
Alharbi Edward [93]	2020	Saudi Arabia	1	29	To report a case of VKC with Rosai-Dorfman disease	The association of RDD with VKC has not been previously reported; however, the causal relationship remains unclear	High
Bajracharya et al. [94]	2020	Nepal	1	19	To report a case of sensory exotropia due to pellucid marginal degeneration (PMD) in association with VKC	A child with VKC should undergo regular refraction so as not to miss any ectatic changes occurring in the cornea	High
Farias et al. [95]	2021	Brazil	1	8	To describe an 8-year-old boy unsuccessfully treated for years for VKC	Only after antiretroviral treatment, the allergy symptoms completely regressed. Consider investigation of HIV infection in patients with refractory allergic conjunctivitis	High
Fukushima Tabuchi [96]	2022	Japan	1	11	To describe a case of VKC associated with concomitant growth hormone deficiency (GHD)	When treating VKC patients, it is important to bear the likelihood of GHD in mind, because of the possible correlation with VKC signs and symptoms	Medium
Artesani et al. [97]	2023	Italy	1	17	To describe a case of VKC associated with down syndrome (DS)	Finding an inflammatory/allergic disease such as VKC in DS is unusual, but it must be taken into account because keratoconus, one of the most frequent eye pathologies in DS, can be secondary to an unrecognized VKC	Medium

*N/A* non-available information

**Table 8:** Therapy systematic reviews

Author	Year	Country	No. of VKC studies	N VKC patients	Median age	Outcome	Results	Amstar score
Leonardi et al. [20]	2019	Italy	3	1205	13.7	To provide a review of the currently available treatments for ocular allergy	Topical antihistamines, mast cell stabilizers, or double-action drugs are the first choice of treatment. Topical calcineurin inhibitors may be used in steroid‐dependent or resistant cases of severe allergic keratoconjunctivitis, like VKC	Low
Singhal et al. [98]	2019	India	30	3915	N/A	To evaluate current options in VKC therapy	Most cases of VKC can be managed with medication alone. Surgical therapy may be used in case of severe giant papillary hypertrophy or shield ulcer	Low
Roumeau et al. [99]	2021	France	45	1749	11.2	To evaluate the efficacy of medical treatments for VKC	Mast cell stabilizers are useful in milder forms. The efficacy of cyclosporine and tacrolimus is similar, suggesting that tacrolimus is a good alternative to cyclosporine for severe cases of VKC	High
Rasmussen et al. [100]	2022	Denmark	39	2046	13.1	To systematically review the literature on the treatment of VKC	Topical corticosteroids are the most effective therapy for VKC	High

**Table 9:** Therapy narrative reviews

Author	Year	Country	No. of VKC studies	N VKC patients	Median age	Outcome	Results	SANRA scale
Esposito et al. [101]	2016	Italy	N/A	N/A	N/A	To review treatment of VKC	Moderate to severe VKC should be treated with cyclosporine or tacrolimus eye drops. However, there is no worldwide consensus on VKC treatment	Medium
Gokhale [23]	2016	India	N/A	N/A	N/A	To assess the best ocular treatment of VKC based on disease severity	Mild VKC should be treated with antihistamines and mast cell stabilizers. Moderate and severe disease should be treated with topical steroids, cyclosporine, and tacrolimus	Low
Kraus [18]	2016	USA	N/A	N/A	N/A	To describe VKC principles of therapy	Antihistamines and mast cell stabilizers are useful in the mildest form of VKC. Moderate-severe forms may require also topical steroids, immunomodulatory agents, and surgical therapy	Critically low
Berger et al. [19]	2017	USA	N/A	N/A	N/A	To propose a treatment algorithm of allergic conjunctivitis (AC)	Topical steroid and allergen-specific immunotherapy could be used as second- and third-line treatment	Medium
Doan et al. [16]	2017	France	3	4	N/A	To describe omalizumab therapy in VKC	Omalizumab reduces signs and symptoms and seems to be a potent treatment for refractory forms of VKC. However, its efficacy is variable among patients	Medium
Takamura et al. [24]	2017	Japan	N/A	N/A	N/A	To review VKC local treatment	The first option is antiallergic eye drops. Moderate-severe forms may require a higher dose of steroid eye drops, or steroid oral therapy	Critically low
Thong [25]	2017	Singapore	4	1800	15.7	To evaluate cyclosporine and tacrolimus efficacy in VKC	Cyclosporine 0.05%, 0.1%, and 1% and tacrolimus 0.1% ophthalmic solutions reduce VKC signs and symptoms	Low
Sacchetti et al. [26]	2018	Italy	N/A	N/A	N/A	To review treatment of allergic conjunctivitis	Cyclosporine and tacrolimus could be used as steroid-sparing agents in the treatment of moderate-severe VKC	Medium
Erdinest et al. [102]	2019	Israel	7	1121	N/A	To present an update of topical tacrolimus for allergic eye diseases	Most patients treated with tacrolimus 0.003–0.1% eye drops showed clinical improvement. Tacrolimus seems to be an effective alternative to cyclosporine eye drops	Medium
Fauquert [27]	2019	France	391	N/A	N/A	To present the salient points concerning the treatment of ocular allergy	First-line treatments are the physical treatments, second line are mast-cell stabilizers and antihistamines, and third-line treatments include local steroids, cyclosporine, immunotherapy, and surgical treatment	Medium
Nebbioso et al. [103]	2019	Italy	4	3198	N/A	To evaluate the use of cyclosporine 0.1% for severe VKC	Cyclosporine 0.1% (Papilock mini® and Verkazia®) administered 2–4 times a day for 4–6 months is effective in controlling VKC signs and symptoms	Medium
AlHarkan [104]	2020	Saudi Arabia	132	N/A	N/A	To review which drugs could be used in Saudi Arabia to treat VKC	Steroid eye drops, CsA 1% and tacrolimus 0.1% eye drops are effective in VKC treatment. However, off-label drugs are not available in Saudi Arabia	Medium
Bielory et al. [28]	2020	USA	N/A	N/A	N/A	To describe the treatment of ocular allergy	Ocular allergy therapy should rely on antihistamines, mast cell stabilizers, steroidal or non-steroidal anti-inflammatory drug (NSAID) eye drops, cyclosporine and tacrolimus eye drops	Low
Di Zazzo et al. [2]	2020	Italy	N/A	N/A	N/A	To describe VKC therapy in adults	The principles of management of childhood and adult VKC essentially remain the same	Low
Stock et al. [17]	2020	Brazil	8	55	11.7	To review surgical debridement of VKC shield ulcer	Surgical debridement is extremely effective in the treatment of shield ulcers. The procedure is followed by a rapid corneal re-epithelialization	Medium
Biermann et al. [105]	2021	Deutschland	N/A	N/A	N/A	To present a treatment plan for severe VKC	Topical CSA is a steroid-sparing agent that permits long-term reduction of exacerbations	Medium
Brindisi et al. [30]	2021	Italy	N/A	N/A	N/A	To improve the management of patients with VKC	There is substantial agreement on the use of cyclosporine as the first-choice therapy. In case of failure, tacrolimus can be used or, if other markers of allergic diathesis (asthma, dermatitis, urticaria) are present, omalizumab can be chosen	Low
Chigbu and Labib [106]	2021	USA	N/A	N/A	N/A	To focus on potential drug targets in VKC	Current and future research should continue to focus on developing immunopharmacological agents that would be beneficial to individuals with VKC	Medium
Feizi et al. [107]	2021	Iran	N/A	N/A	N/A	To describe management of corneal complications in VKC	Corneal transplantation may be required in the advanced stage of keratoconus. Both penetrating keratoplasty and deep anterior lamellar keratoplasty can result in excellent visual outcomes in keratoconus eyes with concomitant VKC	Medium
Ghiglioni et al. [31]	2021	Italy	N/A	N/A	N/A	To describe VKC manifestations	VKC represents, as AKC, a potentially severe and complex disease in its management among the various allergic eye diseases	Medium
Wajnsztajn and Solomon [34]	2021	Israel	N/A	N/A	N/A	To describe treatment of keratoconus	Topical treatment with tacrolimus can significantly reduce the allergic inflammatory response in VKC, reducing the chances of developing keratoconus	Medium
Kaur and Gurnani [35]	2022	India	N/A	N/A	N/A	To summarize the management of VKC	The treatment of vernal keratoconjunctivitis depends on the extent and severity of the disease at the time of presentation. The management may vary from conservative treatment to surgical interventions	Low
Fernandez et al. [108]	2022	USA	N/A	N/A	N/A	To describe new anti-eosinophilic therapies in VKC	Eosinophil pathway mediators like CCR3/CCL11, PGD2/CRTH2, α4β1 Integrin, galectin-1, IL-5/IL-5R, IgE, and Siglec-8 may prove useful therapeutic targets in addressing eosinophilic inflammation found in the conjunctiva	Medium
Mehta et al. [36]	2022	Singapore	N/A	N/A	N/A	To develop recommendations for the management of VKC in Asia	The use of immunomodulators should be considered early to tackle the inflammatory and chronic nature of VKC, with topical corticosteroids reserved as an add-on, short-pulse therapy for persistent disease or corneal involvement	High
Dahlmann-Noor et al. [109]	2023	UK	N/A	N/A	N/A	To provide clear guidance for primary care physicians and ophthalmologists	Patients with signs (“red flags”) indicating severe VKC, or persistent mild-to-moderate VKC that is non-responsive following 2–4 weeks of treatment, should be referred to a sub-specialist	High
Doan et al. [110]	2023	France	17	46	N/A	To evaluate the effects of omalizumab in VKC	Omalizumab treatment is well tolerated with improvement or resolution of ocular symptoms, reduction in steroid use, and enhancement of quality of life	High
Ghauri et al. [111]	2023	UK	N/A	N/A	N/A	To describe best practice recommendations for UK settings	With a consistent and informed approach, adequate information, and local protocols, it is possible to improve the experiences of patients with VKC and achieve consistently high standards of care and clinical outcomes in most clinical settings	High

*N/A* non-available information

**Table 10:** Therapy prospective observational studies

Author	Year	Country	No. of VKC patients	Median age	Outcome	Results	Strobe checklist
Al-Amri et al. [112]	2016	Saudi Arabia	20	23.1	To evaluate the safety and efficacy of tacrolimus 0.1% ointment for refractory VKC	6 weeks of tacrolimus 0.1% therapy permitted a significant improvement in VKC symptoms (*p* < 0.001) and signs (*p* < 0.001)	Medium–low
Barot et al. [113]	2016	India	36	9.3	To study the therapeutic effect of 0.1% tacrolimus eye ointment in patients with allergic ocular diseases	Signs and symptoms significantly decreased after beginning tacrolimus 0.1% ointment treatment (*p* < 0.0001). 36% of patients complained of a transient burning sensation during treatment	Medium–low
Chatterjee and Agrawal [114]	2016	India	23	14.7	To evaluate the efficacy of 0.03% tacrolimus ointment in the treatment of VKC	Symptom and signs significantly reduced at 4 and 12 weeks (*p* < 0.0001). Visual acuity showed an improvement after 12 weeks of treatment (*p* = 0.05)	High
Yücel and Ulus [115]	2016	Turkey	30	12.9	To evaluate the efficacy and safety of topical cyclosporine A 0.05% in the treatment of VKC	Topical CsA 0.05% (Restasis®) was effective in reducing VKC signs and symptoms (*p* < 0.001)	Medium–low
Abozaid [116]	2017	Egypt	11	13.6	To assess the safety and efficacy of femtosecond laser-assisted Keraring implantation followed by transepithelial accelerated corneal collagen cross-linking (CXL) for the treatment of keratoconus in children with VKC	All the eyes treated showed an improvement in visual acuity, keratometry values and refraction (*p* < 0.001). No intraoperative complications were reported	Medium
Al-Amri et al. [117]	2017	Saudi Arabia	20	16.9	To evaluate the safety and efficacy of 0.003% tacrolimus suspension for the treatment of refractory VKC	6 weeks of tacrolimus 0.003% therapy permitted a significant improvement in VKC symptoms (*p* < 0.001) and signs (*p* < 0.001), with no important side effects	Medium–low
Costa et al. [118]	2017	Brazil	17	12.3	To evaluate the use of supratarsal injection of triamcinolone acetonide in severe VKC	Allergy symptoms and signs were significantly improved with the treatment. No side effects were reported	Medium–low
Liendo et al. [119]	2017	Brazil	25	12.0	To evaluate the use of 0.03% topical tacrolimus in severe allergic keratoconjunctivitis	Topical tacrolimus significantly decreased VKC signs and symptoms (*p* < 0.001). It could be an effective therapeutic option for severe ocular allergy	High
Wan et al. [120]	2018	China	17	N/A	To describe the effect of topical 0.1% tacrolimus eye drops in VKC	After 1 week, there were significant reductions in VKC signs (*p* < 0.001) and symptoms (*p* < 0.001)	Medium
Maitra et al. [121]	2018	India	248	8.2	To assess the drug usage pattern for the management of VKC	26 different formulations were prescribed. The lower grades were treated with anti-allergic and lubricant drops. Low potency steroids were prescribed in very early grades of VKC	Medium
Fiorentini and Khurram [122]	2019	United Arab Emirates	10	N/A	To evaluate the use of topical 0.03% tacrolimus ointment for VKC in the Middle East	4 weeks of tacrolimus therapy permitted a significant reduction in signs and symptoms, without side effects	Low
Samyukta et al. [123]	2019	India	30	8.2	To evaluate the efficacy of topical tacrolimus 0.03% monotherapy for the treatment of VKC	After 8 months of treatment with tacrolimus 0.03%, VKC signs and symptoms fell drastically (*p* < 0.001), with also an improvement in visual acuity (*p* = 0.04)	Medium
Shoji et al. [124]	2019	Japan	1821	19.3	To evaluate the efficacy of topical 0.1% tacrolimus ophthalmic suspension in chronic allergic conjunctival disease with and without atopic dermatitis	Tacrolimus therapy decreased ocular signs and symptoms in both groups (*p* < 0.0001). The concomitant use of topical steroids significantly increased the likelihood of remission (*p* < 0.0001)	Medium
Xu and Cai [125]	2019	China	926	15.1	To evaluate therapeutic effects and safety of houttuynia eye drops combined with olopatadine hydrochloride eye drops on VKC	A combination of houttuynia eye drops and olopatadine permitted a reduction in VKC symptoms (*p* < 0.05), without side effects	Low
Modugno et al. [126]	2020	Italy	23	18.4	To compare corneal morphologic changes in VKC patients treated with topical cyclosporine	Cyclosporine therapy caused corneal microstructural changes at the level of epithelium, sub-basal nerve plexus, and stroma (*p* < 0.001), helping to restore the normal corneal microstructure	Medium–low
Heikal et al. [127]	2022	Egypt	59	9.5	To compare the effects of cyclosporine A (2%) eye drop and tacrolimus (0.03%) eye ointment on children with VKC not responding to corticosteroid eye drops	Individual symptoms and signs were significantly reduced in the tacrolimus group compared to those in the cyclosporine A group (*p* < 0.05, *p* = 0.037)	High
Malhotra et al. [128]	2021	India	38	15.1	To compare the efficacy of 2% rebamipide suspension with topical cyclosporine and tacrolimus for managing VKC	The reduction of mean sign scores between rebamipide and tacrolimus and between rebamipide and cyclosporine was comparable	High
Sruthi et al. [129]	2021	India	50	N/A	To evaluate the effectiveness and safety of olopatadine 0.1% ophthalmic drops with bepotastine besilate 1.5% ophthalmic drops in patients with VKC	Bepotastine eye drops proved quicker relief of symptoms and signs compared to olopatadine	Medium
Bourcier et al. [130]	2022	France	46	8.8	To compare efficacy and safety of 0.1% cyclosporine vs 2% cyclosporine in the treatment of severe VKC	An improvement in symptomatic and clinical scores was observed, regardless of cyclosporine posology. There was no difference in progression between the two concentrations	Medium
Pradhan et al. [131]	2022	India	63	8.8	To evaluate the effectiveness of a modified therapeutic protocol used for VKC based on severity as per Bonini grading system	70% showed signs of significant improvement in grade by the end of 6 weeks, reaching 90% at the end of 6 months (*p* = 0.074) and 92% at the end of 12 months (*p* = 0.002)	Medium
Tanaka et al. [132]	2022	Japan	2	N/A	To describe the use of Antihistamine-Releasing Contact Lenses (ARCL) in ocular-allergic conjunctivitis	Following the use of ARCL, six patients were satisfied. However, ARCL should be introduced after allergic conjunctivitis is controlled or becomes asymptomatic	Medium
Giannaccare et al. [133]	2023	Italy	25	8.4	To report the clinical outcomes of topical 0.1% ciclosporin cationic emulsion (CsA-CE) in VKC	Symptomatic and clinical scores decreased significantly after treatment (*p* < 0.0001). Five patients (20%) required at least one course of rescue medication (mean of 3.4 ± 4.8 courses/year)	High
Mohan et al. [134]	2023	India	221	N/A	To explore the efficacy of olopatadine 0.1% treatment for VKC	Olopatadine 0.1% twice a day permits relief in subjective symptoms of itching, tearing, and redness (*p* < 0.01)	High

*N/A* non-available information

**Table 11:** Therapy retrospective observational studies

Author	Year	Country	No. of VKC patients	Median age	Outcome	Results	Strobe checklist
Shoughy et al. [135]	2016	Saudi Arabia	62	12.0	To evaluate the efficacy and safety of topical low-dose tacrolimus (0.01%) solution in patients with VKC	Tacrolimus 0.01% ophthalmic solution permitted significant improvement in VKC symptoms (*p* < 0.001) and signs (*p* < 0.001)	High
González-Medina et al. [136]	2018	Spain	17	12.0	To evaluate the usefulness and safety of topical tacrolimus 0.03% ointment in VKC children	0.03% tacrolimus eye ointment allowed for the cessation of antihistamines therapy in 8 patients (*p* < 0.05). The number of flare-ups per year was not reduced, but the duration and the severity of each exacerbation were reduced	Critically low
Iyer et al. [137]	2018	India	6	10.6	To report outcomes of mucous membrane grafting (MMG) for refractory giant papillae in VKC	After MMG, reactivation of the allergic activity was noted in all eyes, but with no recurrence of shield ulcers or diffuse punctate keratitis. Giant refractory papillae could be an indication for early surgical excision with MMG	Low
Abozaid et al. [138]	2019	Egypt	28	14.3	To evaluate femtosecond laser-assisted intrastromal corneal ring segments’ (ICRS) implantation followed or accompanied by transepithelial accelerated corneal collagen cross-linking (TE-ACXL) as a treatment of keratoconus in VKC	Better visual acuity (*p* = 0.001) and corneal measure (*p* < 0.001) in patients treated with Keraring + CXL than patients treated with CXL only. The combined ICRS and CXL can improve the visual, refractive, and tomographic parameters	Medium
Alrobaian et al. [139]	2019	Saudi Arabia	19	15.8	To determine the relative safety and efficacy of corneal collagen cross-linking (CXL) in patients with keratoconus and VKC	No significant difference between the baseline and last follow-up in visual acuity (*p* = 0.99) and keratometry values (*p* = 0.093). 5 of 27 eyes with VKC exhibited progression of keratoconus (18.5%)	High
Müller et al. [140]	2019	Brazil	21	12.0	To assess the compliance, efficacy, and safety of the long-term use of topical tacrolimus in VKC	Topical tacrolimus 0.03% ointment permitted to achieve disease control without the use of steroids in 10 (47.6%) patients	High
Liu et al. [141]	2019	Taiwan	10	10.5	To evaluate the effects of tacrolimus ointment to treat refractory VKC	Tacrolimus 0.1% ointment reduced conjunctival and corneal reaction (*p* = 0.0003 and 0.0002). In 6 out of the 10 patients, tacrolimus treatment enabled discontinuation of steroid therapy (*p* < 0.05)	High
McSwiney et al. [142]	2019	Ireland	25	9.1	To describe treatment with supratarsal injection of triamcinolone for VKC	Supratarsal injections of triamcinolone acetonide 15–30 mg in VKC patients (1–9 injections) led to an improvement in visual acuity (*p* < 0.0001). 100% of patients had improvement in symptoms	Medium
Sen et al. [143]	2019	India	47	14.1	To describe management and outcome of steroid induced glaucoma in VKC	Intraocular pressure (IOP) was controlled by the withdrawal of steroids, antiglaucoma medications, trabeculectomy. With the treatment, there was a statistically significant reduction in IOP (*p* < 0.00001)	High
Jongvanitpak et al. [83]	2020	Thailand	20	7.9	To describe outcomes of treatment in children with ocular allergy	68.8% of VKC patients used topical steroids to control the disease. Subcutaneous immunotherapy was performed in 2 patients after using tacrolimus eye ointment with benefits	High
Caputo et al. [144]	2021	Italy	431	8.5	To evaluate the safety and efficacy of tacrolimus 0.1% eye drops in refractory VKC	All the clinical signs significantly improved during the whole follow-up in both the perennial and seasonal forms	High
Elubous et al. [145]	2021	Jordan	20	31.2	To identify environmental risk factors associated with the need for penetrating keratoplasty (PKP) in patients with keratoconus	VKC is a statistically significant risk factor (*p* = 0.005) for PKP	High
Feizi et al. [146]	2021	Iran	117	25.4	To compare outcomes after penetrating keratoplasty (PK) against deep anterior lamellar keratoplasty (DALK) for keratoconus in patients with VKC	There is no difference in outcomes between PK and DALK for keratoconus in patients with VKC	High
Gupta et al. [147]	2021	India	50	8.3	To compare the efficacy of eye drop interferon (IFN) α-2b with tacrolimus 0.03% in refractory VKC	IFN α-2b results in greater improvement in subjective symptoms and objective signs, has fewer side effects in long term, and is better tolerated as compared to tacrolimus	High
Hirota et al. [148]	2021	Japan	17	20.0	To evaluate the clinical improvement and safety of prolonged treatment of VKC and AKC using topical tacrolimus	Two years of treatment with topical tacrolimus ophthalmic suspension is an effective method for inducing and maintaining the stable stages of VKC and AKC	High
Maharana et al. [149]	2021	India	11	13.7	To describe the role of combined topical cyclosporine 0.1% and tacrolimus 0.03% in cases of severe steroid intolerant VKC	Combined use of cyclosporine and tacrolimus may lead to rapid resolution of symptoms and reduced recurrence rate in cases with severe VKC in which a steroid has to be avoided	High
Yazu et al. [150]	2021	Japan	5	15.7	To evaluate the long-term outcomes of using 0.1% tacrolimus eye drops to treat severe allergic conjunctival diseases	Topical tacrolimus may provide effective and long-term improvement in clinical signs of severe AKC and VKC cases that are refractory to standard conventional treatment	High
Arnon et al. [151]	2022	Israel	85	7.8	To compare treatment regimens of tacrolimus and of topical steroids for VKC and suggest a treatment protocol	Tacrolimus as 1st line treatment may be preferred for severe cases, for faster disease remission compared to tacrolimus as 2nd line treatment, and with fewer topical treatments per day compared to topical steroids	High
Salami et al. [152]	2022	Italy	29	N/A	To report the clinical outcomes of topical 0.1% ciclosporin cationic emulsion (CsA-CE) in VKC	CsA-CE was effective in reducing signs and symptoms in daily clinical practice. 55% of treated patients required the additional use of a 3-day course of topical dexamethasone with 1.13 ± 0.81 mean courses/month	High
Senthil et al. [153]	2022	India	82	15.8	To report outcomes and assess the risk factors for failure of trabeculectomy, trabeculectomy with mitomycin‑C, and combined trabeculectomy with cataract extraction in VKC eyes with steroid-induced glaucoma	The surgical success for all three types of surgery is similar at 5 years. Chronic VKC and long‑term steroid use are associated with surgical failure	High
Arora et al. [154]	2023	India	3	17.0	To evaluate the effectiveness of repeat deep anterior lamellar keratoplasty (DALK) in patients of previous failed DALK	Best-corrected visual acuity improved from 20/120 to 20/30 at the end of 1‑year post repeat DALK in all except one patient	Medium
Priyadarshini and Das [155]	2023	India	N/A	N/A	To elicit opinions on the preferred practice pattern in the treatment of allergic eye disease	Dual-acting agents are preferred by 40% in mild-moderate clinical variants. Topical steroids in slow tapering dosage are preferred by 86.7% of ophthalmologists	Medium
Rashid et al. [156]	2023	South Africa	N/A	N/A	To explore keratoconus diagnosis and management in Kenya	Few practitioners had access to a corneal topographer (13.5%; *p* = 0.08). Corneal topography was not recommended in two-thirds of patients (59.0%; *p* = 0.33) with VKC	High
Saha et al. [157]	2023	India	36	7.7	To compare the efficacy and safety of tacrolimus 0.03% and 0.1% eye ointment in the treatment of recalcitrant VKC	Both strengths of tacrolimus (0.03% and 0.1%) are effective in recalcitrant VKC. Papillae respond better with higher strength (0.1%) but is associated with more significant side effects	High

*N/A* non-available information

**Table 12:** Therapy cases series

Author	Year	Country	No. of VKC patients	Median age	Outcome	Results	JBI checklist
Heffler et al. [158]	2016	Italy	2	N/A	To describe the usage of omalizumab in VKC patients, not affected by asthma	With the monthly administration of omalizumab, both patients had an improvement in VKC symptoms, physical examination, and conjunctival cytologic findings. Omalizumab was an effective treatment in patients with VKC without concomitant asthma	Critically low
Doan et al. [16]	2017	France	4	9.2	To describe the usage of omalizumab in severe VKC children	Omalizumab was administered every 2 weeks for 8 weeks. 3 of 4 patients responded to the treatment, but the response was incomplete	Low
Occasi et al. [159]	2017	Italy	4	8.5	To report 4 cases of VKC treated with omalizumab	After a 6-month omalizumab therapy, all children experienced an improvement of ocular symptoms and signs. No relapse was observed after treatment suspension	Low
Callet et al. [160]	2018	France	2	8.0	To describe omalizumab use in VKC and asthma patients	Monthly omalizumab therapy permitted VKC and asthma control in both patients	Low
Westland et al. [161]	2018	Netherlands	3	11.0	To describe an intense regimen of 0.05% cyclosporine for vernal shield ulcers	Cyclosporine 8 times a day treatment provided quick resolution of the shield ulcers and complete re-epithelialization	Low
Maharana et al. [149]	2021	India	11	13.7	To describe the combined topical cyclosporine (CsA) 0.1% and tacrolimus 0.03% use in VKC	Combined therapy allowed an improvement in VKC signs and symptoms (*p* < 0.001)	High
Stock et al. [17]	2020	Brazil	2	5.5	To report two cases of corneal shield ulcer treated which surgical debridement	In both cases, surgical debridement was curative and definitive in the 7-month follow-up period. Shield ulcer did not recur	Medium
Patil and Mehta [162]	2022	Singapore	4	9.8	To report the long-term outcomes of patients with refractory VKC who underwent surgical excision of giant papillae with mitomycin C 0.02% and amniotic membrane transplantation (AMT)	Surgical excision of GP in combination with mitomycin C and AMT, in refractory VKC, is a good treatment option with better clinical outcomes over a longer follow-up	Medium

*N/A* non-available information

**Table 13:** Therapy case reports

Author	Year	Country	No. of VKC patients	Age	Outcome	Results	JBI checklist
Das et al. [163]	2016	India	1	22	To describe the case of a VKC patient successfully treated with local pharmacologic therapy and surgery	Amniotic membrane transplantation, followed by cataract surgery and optical prosthetics, was efficacious in the treatment of VKC complications	High
Agarwal et al. [164]	2018	India	1	9	To describe the case of a 9-year-old boy with HIV who developed also VKC	Systemic steroids or immunosuppression is not recommended in a HIV-positive patient. There is also a correlation between heightened allergic response and progression of HIV disease with decreasing CD4 counts	High
Santamaría and Sánchez [165]	2018	Colombia	1	15	To describe omalizumab use in VKC	The bi-weekly use of omalizumab proved effective in the treatment of VKC. However, upon discontinuation of the drug, the symptoms resumed	High
Simpson and Lee [166]	2018	Canada	1	54	To describe a single dose omalizumab treatment	A single injection of omalizumab resolved VKC signs and symptoms in the adult patient	Medium
Borrego-Sanz et al. [167]	2019	Spain	1	10	To describe the use of oral cyclosporine in VKC	Daily oral cyclosporine therapy allowed the re-epithelialization of vernal shield ulcer and the tapering of steroid eye drops	High
Hopen et al. [168]	2019	USA	1	8	To report a case of intraocular pressure (IOP) reduction after a gonioscopy-assisted transluminal trabeculotomy in a VKC child	Vision and IOP showed an improvement. The only adverse effect was a small hyphema	High
Kurtul and Koca [169]	2021	Turkey	1	3	To report giant papilla appearing as prolapsed mass extending from the tarsal conjunctiva	Topical medical treatment with a steroid and antiallergic eye drops can produce a quick recovery from a giant papilla without the need for surgery	Medium
Özkaya et al. [170]	2021	Turkey	1	12	To present a case of corneal shield ulcer treated with topical cyclosporine A (CsA) and corneal debridement	Shield ulcers should be treated aggressively. The combination of topical CsA and surgical debridement is the most appropriate treatment for grade 2–3 shield ulcers	Medium
Singh et al. [171]	2021	India	1	17	To report a novel technique of doughnut amniotic membrane transplantation (AMT) along with penetrating keratoplasty (PK) for limbal stem cell deficiency in VKC	AMT with or without keratoplasty is a simple and an effective modality of treatment for limbal stem cell deficiency	Medium
Jain et al. [172]	2022	India	1	22	To describe the management of bilateral limbal stem cell deficiency (LSCD) in VKC with allogeneic simple limbal epithelial transplantation (allo-SLET)	The patient underwent a cadaveric allo-SLET in the right eye to restore the ocular surface. Systemic immunosuppression with oral cyclosporine was administered. The corrected visual acuity was 20/20 in both eyes. No recurrence of LSCD was observed	High
Kate et al. [173]	2022	India	1	32	To describe the clinical features and management in VKC with bilateral tarsal conjunctival keratinization	The patient underwent excision of the conjunctival keratinization in both eyes. The resultant bare areas were covered with conjunctival autografts	High
Shih et al. [174]	2022	Taiwan	1	18	To report the case of an 18-year-old male patient presenting with acute onset of corneal hydrops and refractory steroid-induced glaucoma	Tacrolimus dermatological ointment showed satisfactory efficacy when combined with topical cyclosporine and steroid, without VKC reactivation	Medium

*N/A* non-available information

**Table 14:** Therapy randomized clinical trials

Author	Year	Country	No. of VKC patients	Median Age	Outcome	Results	Jadad score
Gayger Müller et al. [175]	2017	Brazil	16	11.6	To evaluate tacrolimus versus sodium cromoglycate monotherapy in VKC	Tacrolimus was more effective than sodium cromoglycate in controlling VKC signs and symptoms (*p* = 0.001 and 0.015)	High
Zanjani et al. [176]	2017	Iran	40	10.9	To compare tacrolimus and interferon alpha-2b (IFN alpha-2b) eye drops in the treatment of VKC	Both patients treated with tacrolimus 0.005% or IFN alpha2b eye drops showed an improvement in VKC signs and symptoms after 3 years (*p* < 0.0001 for both groups), without significant statistical difference between the two groups (*p* > 0.05)	High
Leonardi et al. [177]	2019	Italy	169	9.2	To evaluate the efficacy and safety of cyclosporine A cationic emulsion (CsA CE) in the treatment of VKC	Patients treated with CsA CE showed a higher improvement in VKC signs and symptoms rather than placebo (*p* = 0.007 and 0.010) and had less use of rescue steroid eye drops (*p* = 0.010 and 0.055, respectively)	High
Bremond-Gignac et al. [178]	2020	France	142	9.1	To assess the safety and efficacy of cyclosporine A cationic emulsion (CsA CE) 0.1% eye drops in VKC	The most common adverse events were instillation site pain and pruritus. The reductions in signs and symptoms achieved with CsA CE during the 4-month evaluation (Leonardi 2019) period were maintained during the 8-month follow-up period	High
Iqbal et al. [179]	2020	Egypt	38	14.3	To compare standard epithelium-off cross‐linking (SCXL) versus accelerated epithelium‐off cross‐linking (ACXL) and transepithelial epithelium‐on cross‐linking (TCXL) in the treatment of keratoconus (KC) in children	There were significant differences in visual acuity and refractive measure between the three groups throughout the study (*p* < 0.0001) in favor of SCXL followed by ACXL. SCXL protocol was superior to ACXL and TCXL, with an overall success rate of SCXL being 100% during 2 years of follow-up	Medium
Chen et al. [180]	2021	China	76	9.9	To compare the efficacy of the combination of 0.05% azelastine and 0.1% tacrolimus eye drops with 0.1% tacrolimus monotherapy in VKC	The combination of 0.05% azelastine and 0.1% tacrolimus eye drops leads to faster and greater improvements in clinical signs and symptoms	Medium

*N/A* non-available information

### Quality Assessment

All the studies considered in the following review were analyzed to evaluate their clinical significance according to appropriate scales. The quality of the eligible studies was evaluated using different methods according to the study design: the Amstar 2 Checklist for Systematic Reviews [11], the SANRA scale for Narrative Reviews [12], the Jadad score for Randomized Clinical Trials (RCT) [13], the Strobe Checklist for the Observational Studies [14], the Joanna Briggs Institute (JBI) Critical Appraisal Checklist for Case Reports [15], and the Joanna Briggs Institute (JBI) Critical Appraisal Checklist for Case Series [15]. From each study, we considered information regarding study design, date of publication, country of origin, setting, characteristics of the population sample, objective of the study, and outcome measure.

None of the systematic reviews fulfils all the characteristics required. Meta-analysis was performed only in Rasmussen et al. [22, 100] and Roumeau et al.’s study [99]. Regarding Leonardi et al.’s therapeutic review [21], this study performed excellent systematic research in the literature using multiple databases and selected all the articles with two separate reviewers. However, the included studies were not described in detail, and a complete list of the excluded studies was not presented. The authors did not use a satisfactory technique for assessing the risk of bias. As regards Leonardi et al.’s [20] therapeutic review, also this study performed excellent systematic research in the literature, although there was not a study selection and extraction in duplicate. There was also a lack of assessing the risk of bias and in providing a list of the excluded studies. The systematic review of Singhal et al. [98] performed good systematic research in the literature, but the research was not carried out by separate reviewers. Like Leonardi et al. [20], this review did not provide a complete list of the excluded studies and did not use a technique for assessing the risk of bias. However, all the included studies were described indicating populations, interventions, comparators, outcomes, and research designs.

Roumeau et al. [99] and Rasmussen et al. [22, 100] conducted a detailed meta-analysis of the literature. One author conducted all literature searches and collated the abstracts. Two authors separately reviewed the abstracts and, based on the selection criteria, decided on the suitability of the articles for inclusion. The risk of bias is described in detail. The two systematic reviews of Rasmussen et al. [22, 100] performed an excellent meta-analysis of the literature and described in detail the risk of bias. However, both Roumeau et al. [99] and Rasmussen et al. [22, 100] do not provide a list of the excluded studies.

Narrative reviews were evaluated through The SANRA scale [12]. None of the studies fulfils all the criteria. Only about half of the studies reported information on how the literature search was conducted. The aim of the study was not clearly expressed in six of the narrative reviews. Four studies were found to lack data description. The best performance was attained by Dahlmann-Noor et al. [109] and Doan et al. [110] (11/12), followed by Mehta et al. [36] and Ghauri et al. [111] (10/12). Except for Singh et al. [33], they all performed excellent literature research, detailing search terms and inclusion criteria. In all these works, key statements are supported by adequate references. Stock et al. [17] performed the best data presentation. On the other hand, the worst performance was of Takamura et al. [24] and Kraus [18] (1/12 and 2/12, respectively). Both articles lack a justification of the article’s importance, no concrete aims or questions were expressed, the search strategy was not presented, and data were presented inadequately. In Kraus’s work [18], appropriate evidence was introduced selectively, while in Takamura et al. [24] the article’s point was not based on appropriate arguments.

Randomized clinical trials were evaluated using the Jadad scale [13]. All of them obtained a minimum of 3/5 points.

Leonardi et al. [177], Bremond-Gignac et al. [178], and Gayger Müller et al. [177] fulfil all the checklist criteria. In the trial conducted by Zanjani et al. [176], the randomization model was not described in detail. The study of Chen et al. [180] did not mention how the blinding was performed.

Observational studies were analyzed using the Strobe Checklist [14]. The only study that reached the maximum score was that of Zhang et al. [62]. All the retrospective and observational diagnosis studies included in their works an appropriate abstract, an introduction with the literature underground, the rationale, and the design of their studies. However, in five cases, the context of the study was not described in detail and in three cases were not reported all the relevant dates (recruitment, exposition, follow-up), while in two cases were not cited the setting and hospital in which the study was conducted. All the studies described the inclusion criteria and how the patients were included, but only six of them described how they arrived at the final sample size. Only one study described how the authors managed the confounding factors and the risk of bias (Horinaka et al. [52]). Only seven studies described how they managed the quantitative variables in the analysis. One study (Gupta et al. [79]) did not include in its methods an accurate statistical analysis description. In the discussion section, all the studies described their results, an interpretation of them based on the available literature and a generalization of the results they achieved. However, eighteen studies did not report the limits of their works. Also, all the retrospective and observational therapy studies included in our research had an appropriate abstract (except for González-Medina et al. [136]), an introduction with also the rationale of their works, and a description of how the study was managed. Six articles did not report all the relevant dates, and in one case, the setting was not described. One study (González-Medina et al. [136]) did not report the inclusion criteria for patients’ recruitment. Most of the studies did not explain how they arrived at the final sample size. Only three authors considered the risk of bias in their analysis (Liendo et al. [119], Müller et al. [140], and Feizi et al. [146]). In ten cases, the statistical methods used in their works were not described in detail. In five studies, there was a lack in the demographic description of the patients enrolled. One study (González-Medina et al. [136]) did not discuss the results achieved. Fifteen studies did not report the limits of their works. In one study (Samyukta et al. [123]), the funding sources were not reported.

Case reports and case series were analyzed through the Joanna Briggs Institute (JBI) Critical Appraisal Checklist [15].

None of the case reports completely fulfilled the checklist criteria. In fact, in all the articles, we found missing information about the patient (in particular, ethnicity and anamnestic history). In six articles, it was not reported if the patient developed any adverse effects from the drugs used. In four works, information about how VKC signs were evaluated was missing.

Similarly, none of the analyzed case series completely fulfilled the checklist criteria, scoring 9 out of 10 on the JBI Critical Appraisal Checklist for Case Series [15]. Two studies (Maharana et al. [149], Patil et al. [162]) described inclusion criteria in detail. Only one study (Maharana et al. [149]) performed a consecutive inclusion of the participant, although not all the consecutive patients were included in the study, and performed a statistical analysis of the results that emerged.

In all the cases series, except Stock et al. [17], we found missing information about the patients (particularly race). In two cases, VKC signs and symptoms were not measured in a standard, reliable way (Heffler et al. [158], Callet et al. [160]). In one case (Heffler et al. [158]), it was not described how VKC diagnosis was performed and there was missing information about the patients enrolled.

## Clinical Manifestations of VKC

The children affected by VKC come to the ophthalmologist and pediatrician’s attention complaining of intense itching and conjunctival hyperemia. They usually show intense photophobia, white mucous discharge (particularly in the morning), and foreign body sensation. In most severe cases, there is also a burning sensation or ocular pain, suggestive of corneal involvement [1].

None of the symptoms complained by the patients (itching, photophobia, foreign body sensation) is pathognomonic of VKC, but in other ocular allergies, like seasonal allergic conjunctivitis or perennial allergic conjunctivitis, these symptoms are usually milder than in VKC.

**Fig. 4:**
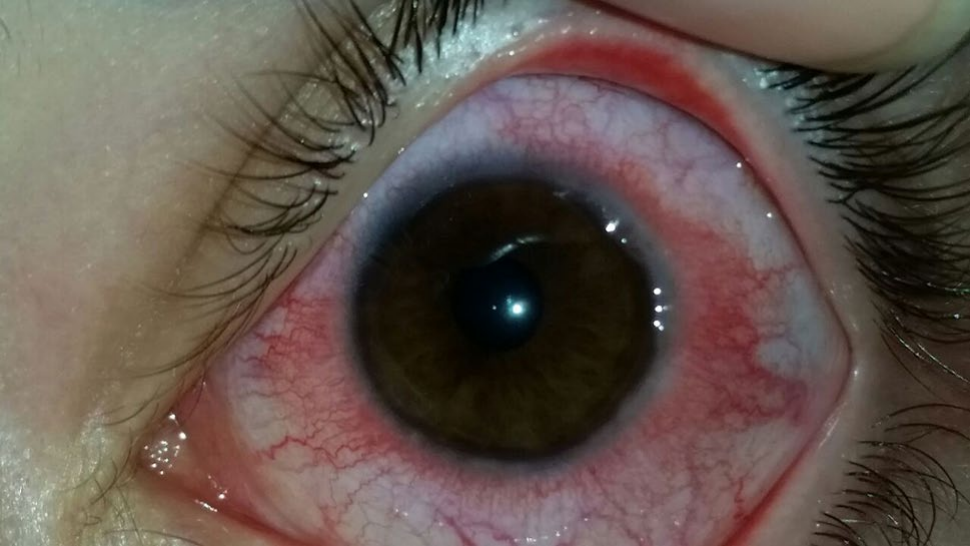
Conjunctival hyperemia and Trantas dots present in VKC patients

**Fig. 5:**
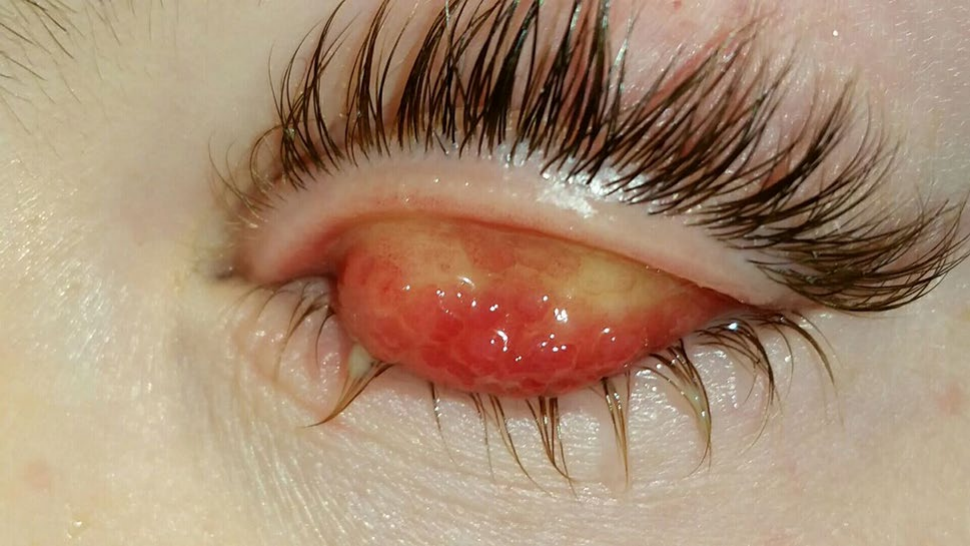
Papillae at the upper tarsal lid present in VKC patients

**Fig. 6:**
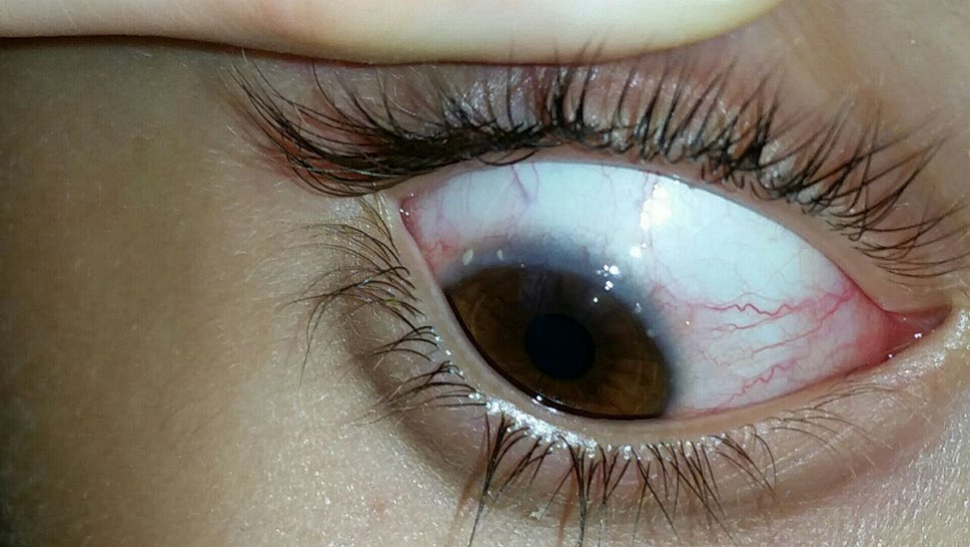
Trantas dots present in VKC patients

VKC patients typically present conjunctival hyperemia (Table 15, Fig. 4), papillae at the upper tarsal lid (Fig. 5), limbal inflammation, and Trantas dots (Figs. 4 and 6) [1]. Other findings are corneal neovascularization and the formation of the so-called “pseudogerontoxon” [8]. If not adequately treated, the disease could evolve into corneal damage, like superficial punctate keratitis and shield ulcers, a pathognomonic sign of VKC [7].

**Table 15:** Main finding in VKC

Conjunctival hyperemia
Tarsal papillae
Trantas dots
Limbal neovascularization
Punctate keratitis

A possible feared complication of VKC, especially in developing countries, is steroid-induced glaucoma. Long-term therapy with steroid eye drops or systemic steroidal drugs may lead, particularly in “steroid-responder” patients, to a progressive increase in intraocular pressure (IOP) and glaucoma [1]. An Indian study by Senthil et al. [82] described a prevalence of steroid-induced glaucoma in VKC patients of 2.24%. In these subjects, IOP was medically controlled in 66% of cases, while 34% required surgical treatment. Gupta et al. [79] observed that among the 1259 patients followed in their clinic for active glaucoma, 4% had been prescribed topical steroids for VKC. In these subjects, IOP was medically controlled in 55% of cases, and 45% required filtering surgery.

Another dramatic complication is the development of keratoconus. Ahmed et al. [50] reported keratoconus in 34% of cases of VKC. According to Kavitha et al. [53], all children affected by VKC should be screened for keratoconus, since they have significantly higher posterior corneal elevation than controls. Furthermore, Yılmaz et al. [61] found an increased incidence of posterior corneal astigmatism in VKC cases compared to age- and gender-matched controls.

## VKC Diagnosis

### Ophthalmological Evaluation

At the slit lamp exam, the typical findings of VKC are conjunctival hyperemia, papillae at the upper tarsal lids, and gelatinous infiltration of the limbus and Trantas dots. Papillae are extremely variable in dimensions: in fact, they could range from a few millimeters to giant papillae (> 7–8 mm), giving the tarsal conjunctiva a “cobblestone” aspect [1]. In more severe cases, if the cornea is involved in the inflammatory process, the examination with fluorescein stain could also highlight superficial punctate keratitis and shield ulcers [7].

To assess corneal damage, various scales have been proposed in the last year, such as the Oxford grading system [181] and the modified Oxford scale [181], currently used in patients with dry eye. The last scale was proposed by Leonardi et al. in 2020 and was called “penalties-adjusted corneal staining score” [21]. In this scale, Leonardi et al. proposed to use the change in corneal staining with fluorescein (CFS) from baseline in the modified Oxford scale, with the possibility of penalties in case of rescue therapy or corneal ulcer. In patients whose corneal damage was, according to the Oxford scale, at its maximum level (grade 5), any worsening of corneal damage could not be reported. To capture that further aggravation during follow-up, Leonardi et al. proposed to add a penalty to the score, as follows: + 1 point penalty for rescue medication and + 1 point penalty for corneal ulceration. The “penalty-adjusted corneal staining score” appeared to be a reliable method for assessing corneal changes over time and for evaluating the efficacy of new drugs.

Other findings of VKC are also corneal neovascularization and the so-called “pseudogerontoxon,” characteristic lipid deposition in the limbus [8].

Based on the clinical finding at the ophthalmological exam, VKC can be classified into three forms [7]:

- Tarsal VKC: characterized by the presence of papillae at the upper tarsal lid
- Limbal VKC: characterized by the presence of gelatinous infiltrate at the limbus and Trantas dots
- Mixed VKC: characterized both by the presence of papillae and limbal involvement

Recently, Soleimani et al. [91] observed in VKC older patients a particular clinical finding: the “Splendore-Hoeppli phenomenon.” This phenomenon consists of granulomatous inflammation of the cornea with deposition of eosinophilic material in the conjunctiva. It manifests as multiple yellow lobulated subconjunctival masses with tortuous vessels, usually located at the upper portion of the bulbar conjunctiva, beside the upper eyelid. According to the author, the Splendore-Hoeppli phenomenon seems to be a later manifestation of VKC, occurring in patients affected by vernal keratoconjunctivitis for some decades.

Thong [25] observed in their Asian VKC populations also the presence of pseudomembrane at the upper eyelids and lower eyelid creasing, the so-called Dennie’s lines. The Dennie-Morgan line is a fold in the skin below the lower eyelid. In some cases, it can simply be a genetic trait, but various studies linked them with allergy sensitization.

Gokhale [23] in his review observed how VKC severity could be defined as mild, moderate-intermittent, moderate-chronic, severe, and blinding based on symptoms and clinical findings. Patients with mild disease complain of itching and conjunctival hyperemia. On examination, they present fine velvety papillae on the upper tarsal lid, but no corneal involvement. The clinical observation of patients with moderate VKC reveals the presence of superficial punctate keratitis, gelatinous infiltrate of the limbus (< 50% of the limbus), and Trantas dots.

In severe disease, there is also evidence of active giant papillae, keratitis, macroerosions of the cornea and severe limbal infiltrate (> 50% of the limbus). Patients with blinding VKC show extremely active large cobblestones, active shield ulcers, severe annular limbal inflammation, limbal stem cell deficiency, and scarring.

### Biomarkers

In the last decade, many studies tried to determine if some biomarkers could help VKC diagnosis, especially when the clinical findings were unclear (Tables 16 and 17).

**Table 16:** Potential biomarkers in tears

IgE
Eosinophils
Histamine
Eotaxin-1 and eotaxin-2
IL-16
Eosinophil cationic protein
Alpha-1-antitrypsin
Osteopontin
Periostin
Vascular endothelial growth factor

**Table 17:** Potential biomarkers using impression cytology

H1 and H4 receptors
CCL24, CCL18, CCL22, CXCL1
IL-1β, IL-6, IL-8, TGFβ-1
Toll-like receptors TLR4 and TLR8
Galectin-3

IgE and eosinophil tear levels were elevated in VKC patients if compared to healthy controls, but high levels were found also in atopic keratoconjunctivitis (AKC) and seasonal (SAC) and perennial conjunctivitis (PAC) [29].

A marker that appeared to be more specific for VKC diagnosis was histamine tear levels. VKC patients revealed twice the histamine levels in tear compared to those in healthy controls. However, histamine tear levels might increase also in other ocular conditions, like *Haemophilus influenzae*’s conjunctivitis [29].

In other studies, eotaxin-1 and eotaxin-2 tear levels were found to increase in VKC patients, but also in AKC subjects. Furthermore, the levels seemed to correlate with disease severity and corneal involvement [29].

In 2020, Shoji [29] demonstrated that tear levels of CCL17/TARC, CCL24/eotaxin-2, and IL-16 in VKC and AKC patients were significantly higher than in patients with other allergic conjunctivitis, like SAC and PAC (*p* < 0.01). Thus, the simultaneous evaluation of these markers could help in making the differential diagnosis between AKC/VKC and SAC/PAC. Eotaxin-1 and eotaxin-2 determination had a high sensibility in VKC diagnosis, but low specificity.

Eosinophils cationic protein (ECP), a marker of eosinophil activation, was increased in tears of VKC and AKC patients and correlated with disease severity. Shoji [29] in his recent review described how in patients with VKC tear ECP and eotaxin-2 levels correlated with disease severity (*p* < 0.01).

Another study observed how alpha-1-antitrypsin levels in tears were lower in VKC rather than in the healthy control group [182]. Other potential biomarkers of VKC could be osteopontin and periostin concentrations in tears. In 2016, Fujishima et al. [39] collected tears from patients with ocular allergic disease to determine the level of periostin in the different forms of allergic conjunctivitis. Their work found significantly high periostin levels in a subject affected by ocular allergies than in allergic patients without conjunctivitis (*p* < 0.05), with maximal levels in AKC and VKC (*p* < 0.001).

However, there is a need for further studies to assess if alpha-1-antitrypsin, osteopontin, and periostin dosage may be useful in VKC diagnosis.

Nebbioso et al. [45] evaluated the concentration of the vascular endothelial growth factor (VEGF) in tear and blood samples from patients with VKC. In their study, they found that VKC patients had higher VEGF levels in tears than healthy controls (*p* < 0.05); however, that difference was not confirmed in the blood (*p* = 0.29).

Another study by Nebbioso et al. [46] evaluated the characteristic of lacrimal film in VKC patients through the tear ferning test (TFT) method. They observed in those subjects a pathological alteration of the lacrimal mucous layer (*p* < 0.001) that returned to baseline after a period of treatment with cyclosporine eye drops. This work underlined the possible usefulness of the tear ferning test in the objective evaluation of tear film and as a marker of disease activity and therapeutic efficacy in patients with VKC. Indeed, some factors may change TFT, which are not fully understood.

Inada et al. [41] used impression cytology to determine the levels of H1 and H4 receptors (H1R and H4R) on the ocular surface of VKC and AKC patients. Levels of H1R and H4R were higher in patients in an active stage of disease rather than in the stable group (*p* < 0.05), without significant differences between the AKC and VKC groups. The determination of H1R and H4R correlated with disease severity. However, it did not allow for making a differential diagnosis between AKC and VKC.

Using impression cytology, Leonardi et al. [48] observed that in VKC conjunctiva, there was an overexpression of several chemokines (CCL24, CCL18, CCL22, CXCL1), proinflammatory cytokines (IL-1β, IL-6, IL-8, TGFβ-1), and genes related to Th2- and Th17-signaling families. Toll-like receptors TLR4 and TLR8, Dectin-1/CLEC7A, mincle/CLEC4E, MCR1, NOD2, and NLRP3 and several of their pathway-related genes were significantly overexpressed in VKC. According to the author, the increased expression of several chemotactic factors and co-stimulatory signals required for T cell activation confirms that VKC is mostly cell-mediated with local eosinophilia. Furthermore, the multiple expression of pattern recognition receptors (PRRs) suggests a role of host–pathogen interaction in VKC development.

Costa Andrade et al. [44] used conjunctival impression cytology to evaluate the expression of galectin-3 (Gal-3) in VKC patients and healthy controls. Gal-3 is a β-galactoside binding protein involved in the pathogenesis of ocular allergy, regulating the eosinophil migration, mast cell activation, and production of local cytokines/chemokines. The study showed a significant increase in Gal-3 expression in the epithelium of VKC patients (*p* < 0.001). Furthermore, Gal-3 expression was significantly reduced in VKC patients treated with steroidal eye drops or tacrolimus eye drops (*p* < 0.001). According to the authors, Gal-3 could serve both as a biomarker of VKC and as a relevant therapeutic target to control the disease.

### Ocular Cytology

In the previous paragraph, some results obtained with impression cytology for the study of markers in VKC have been described. Another way of performing ocular cytology is through the quantification of cells and markers in tears via conjunctival brushing or conjunctival biopsy. Ocular cytology, described for the first time in 1977 by Egbert et al. [183], is applied in the study of a discrete number of ocular diseases, such as dry eye, allergic conjunctivitis, and inflammatory systemic diseases with uveitis (Table 18).

**Table 18:** Main finding in ocular cytology

Mast cells
Lymphocytes B and T
Eosinophils
Neutrophils
Fibroblasts
Epitelial cells

For example, the finding of at least one eosinophil or mast cell (always absent in the conjunctiva of healthy subjects) is an optimal marker of allergic conjunctival disease [184].

A recent study by Bruschi et al. [47] performed with conjunctival brushing showed that untreated conjunctiva of VKC patients was characterized by an elevated number of eosinophils, neutrophils, mast cells, and epithelial cells. These cell counts progressively reduced when the subjects were treated with steroidal or immunosuppressive eye drops.

Nebbioso et al. [103] using impression cytology demonstrated that VKC patients had an increased number of goblet cells in the conjunctiva compared to healthy controls, although that difference was not statistically significant. After a cycle of therapy with cyclosporine eye drops, the density of goblet cells progressively reduced (*p* = 0.044).

### Conjunctival Biopsy

In conjunctival biopsies of VKC patients, elevated numbers of mast cells, lymphocytes B and T, eosinophils, and fibroblasts are described [29] (Table 19).

**Table 19:** Main finding in conjunctival biopsy

Mast cells
Lymphocytes B and T
Eosinophils
Fibroblasts
Hsp chaperones

Leonardi et al. [48] used conjunctival biopsy specimens to assess Heat Shock Proteins (Hsp) chaperone levels in the conjunctiva of VKC patients. These proteins are involved in intercellular communication both in physiological and pathological conditions. This study demonstrated that some Hsp subtypes (specifically Hsp27, Hsp40, Hsp70, and Hsp90) were higher in patient’s conjunctiva than in healthy controls. According to the authors, the understanding of the chaperones’ roles in VKC conjunctiva could open new therapeutic scenarios leading, for example, to the use of specific topical inducers or inhibitors of Hsps for preventing severe eye complications.

However, given the invasiveness of the sampling, conjunctival biopsies are exceptionally used in VKC diagnosis.

### Vitamin D Levels in VKC Patients

In the last years, there was an increased interest in the evaluation of vitamin D levels in VKC patients.

Vitamin D is a prohormone substance regulating a wide range of functions in the human body, including the mineral level of bones and the immune system response. The main source of vitamin D is sunlight exposure. Its deficiency can lead to rickets, increased risk of airway infections, and autoimmune diseases. Serum levels of 25-hydroxyvitamin D (25OHD) below 20 ng/mL, reported in up to 50% of children, indicate a deficiency, while serum levels < 30 mg/dL, reported in 80% of pediatric patients, indicate an insufficient concentration of vitamin D in the blood [185]. Children affected by VKC, because of their remarkable photophobia and the worsening of their symptoms in summer with the solar exposition, tend to avoid sunlight and outdoor activities during spring and summer, thereby possibly increasing the risk of vitamin D deficiency.

Ghiglioni et al. [81] observed that in spring, 81% of VKC children had insufficient 25OHD serum levels (< 30 ng/mL) and 33% had an overt deficiency (25OHD < 20 ng/mL). When the subject was treated with cyclosporine or tacrolimus eye drops during the summer, with an improvement in VKC signs and symptoms and consequent increase in sunlight exposure, there was an increase in vitamin D serum levels. In fact, at the end of summer, 39% of children had still insufficient vitamin D levels, but only 4% had 25OHD < 20 ng/mL.

Zicari et al. [43] and Sorkhabi et al. [59] observed that children affected by VKC had lower levels of vitamin D compared to healthy controls. According to Zicari et al. [43], after 6 months of cyclosporine therapy, these levels increased (*p* = 0.004) but were lower than in healthy controls (*p* < 0.05).

Vitamin D levels appear to be a marker of disease control in VKC patients. A treated subject could receive levels of sun exposure similar to other children, allowing for an improvement in vitamin D serum levels. Instead, when the disease is severe and not adequately treated, vitamin D levels remain low.

## Therapy

At the basis of VKC therapy, there are behavioral rules. Among them, the most useful are [20, 31]

- avoiding contact with aeroallergens, like flowers and plants
- avoiding prolonged sunlight exposure
- wearing solar glasses
- applying cold wraps on the eyes
- using artificial tears that could remove or almost dilute allergens present on the ocular surface
- washing face, hands, and hair frequently, especially before going to sleep

However, behavioral rules and artificial tears alone are not able to control VKC symptoms, except in milder forms.

Drugs that proved their efficacy in the treatment of VKC are topical antihistamines, anti-inflammatory eye drops, steroidal eye drops, cyclosporine and tacrolimus eye drops, and, recently, omalizumab [20].

### Antihistamines and Topical Non-Steroidal Anti-Inflammatory Drugs

Among antihistamines and anti-inflammatory eye drops, the most used molecules are shown in Table 20.

**Table 20:** First line drugs in VKC

**Topical mast cell stabilizers**
• Sodium chromoglycate
• Nedocromil
• Lodoxamide
• Spaglumic acid

**Topical antihistamines**
• Evocabastine
• Emedastine

**Topical mast cell stabilizers + antihistamines**
• Sodium chromoglycate + chlorphenamine
• Ketotifen
• Olopatadine
• Epinastine
• Azelastine

**Topical non-steroidal anti-inflammatory drugs (FANS)**
• Indomethacin
• Ketorolac
• Diclofenac

All these drugs proved their efficacy in the mildest form of VKC. However, only in a few cases, they are able alone to control the disease. Antihistaminic and anti-inflammatory therapy could help in the treatment of VKC, but it frequently requires concomitant therapy with steroidal eye drops or immunomodulatory molecules [20].

Ketorolac and diclofenac eye drops, interfering with prostaglandin E2 and I2 synthesis, reduce itching and conjunctival hyperemia but have no effect on papillae dimensions or corneal lesion repair [186].

### Steroid Therapy

Steroidal drugs are effective in controlling inflammation through various mechanisms:

- reducing leucocyte numbers and activity
- blocking IL-2 production and the consequent clonal expansion of lymphocyte T helper
- blocking fibroblast proliferation
- interfering with cyclooxygenase 2 (COX2) activity and blocking prostanoid synthesis
- interfering with the synthesis of histamine, IgG, and other phlogistic factors

Steroidal eye drops are the gold standard therapy for VKC, but, because of their severe adverse effects (increase in IOP, corneal infections, cataract, and glaucoma), the goal is to control the disease using the lowest dose possible of steroid [20].

In the treatment of VKC, the steroid can be administered in three different ways: eye drops (the commonest way of administration), topical injection in the conjunctiva, and oral medications (major efficacy, but higher adverse effects).

#### Steroid Eye Drops

Steroidal eye drop administration is one of the most useful therapies for VKC. They are always effective. If a patient does not show a clinical response within a few days, he might be affected by an ocular bacterial or viral infection complicating VKC, and he should be promptly referred to an ophthalmologist.

The newest steroidal drug loteprednol [187] appears to be safer than previous generation drugs.

Jongvanitpak et al. observed in his retrospective observational study on Thai children that 68.8% of VKC patients use topical steroids to control the disease [83].

In everyday practice, local steroids are used with significantly different therapeutic schemes varying from a gradual tapering scheme over 2–3 weeks, to short and repeatable 3–5-day cycles, to low-dose prolonged daily administrations after a 1–3-week tapering cycle [20, 31]. The most appropriate choice seems to be the 3–5-day scheme [20, 31].

#### Tarsal Injection of Steroid

In a severe form of VKC, the clinician could consider supratarsal injection of corticosteroid to control VKC signs and symptoms. Dexamethasone sodium succinate, triamcinolone acetonide, and hydrocortisone sodium succinate could be used [20].

In 2017, Costa et al. [118] performed a supratarsal injection of triamcinolone acetonide in 17 children with severe VKC, observing a rapid improvement in VKC signs and symptoms without any adverse reaction.

Similarly, McSwiney et al. [142] performed supratarsal injections of triamcinolone acetonide in VKC patients, with an improvement in visual acuity (*p* < 0.0001) and in VKC symptoms in 100% of cases.

#### Steroidal Systemic Therapy

Oral administration of steroids, although very effective in controlling the disease, is rarely implemented as VKC treatment, because of the high frequency and severity of adverse effects reported.

Because of the long duration of VKC symptoms during the year (5–6 months at least), the use of steroid therapy alone is not feasible as chronic therapy. A 6-month steroidal treatment can cause bacterial superinfection, herpetic keratitis, ocular hypertension, glaucoma, and cataract, as described in 3.5% of treated children [1]. In light of these adverse effects, there has been the development over the past few decades of ophthalmic preparations based on cyclosporine and, more recently, tacrolimus eye drops.

### Immunomodulatory Eye Drops

Cyclosporine has numerous effects on the organism [188]:

- blocks lymphocyte T activation
- stops the production of IL-2 and its receptors
- blocks histamine’s release from basophils and mast cells
- reduces the expression of Human Leukocyte Antigen-II (HLA-II) on the cells

Furthermore, cyclosporine [188]

- interferes with hypersensitivity reactions and mast cell degranulation
- reduces ECP and eosinophil levels in tears
- rapidly controls local phlogosis and acts as a steroid-sparing agent

Differently from a corticosteroid, cyclosporine therapy does not cause cataracts or glaucoma. Its potential side effects, when administered orally, are mainly on the liver and kidney.

However, various studies demonstrated that cyclosporine administered as eye drops is not absorbed into the circulation and consequently does not cause systemic side effects [30, 31]. The only adverse reaction described in the literature is burning at the drops’ instillation [189]. This is due to the pharmaceutical formulation of the compound, in which ethylic acid is also present. However, the burning is always transient, lasting only a few minutes [189].

Being an immunosuppressive agent, it could cause bacterial or viral superinfections, rarely reported in the literature [30].

In the last three decades, cyclosporine has been tested in various formulations (diluted in castor oil or artificial tears) and in various concentrations (2%, 1%, 0.5%, 0.25%) (Table 21). Up to now, it is still not known the minimal effective dose for VKC ocular therapy.

**Table 21:** Cyclosporine formulations in VKC from January 2016 to June 2023

Author	Year	Country	Study design	No. of patients	Median age	Concentration	Results	Adverse effects
Thong [25]	2017	Singapore	Narrative review	1800	15.7	0.05%, 0.1%, 1%	Cyclosporine ophthalmic solution reduced VKC signs and symptoms	Eye irritation
Westland et al. [161]	2018	Netherlands	Case series	3	11	0.05%	Cyclosporine treatment provided quick resolution of the shield ulcers and complete re-epithelialization	-
Nebbioso M [103]	2019	Italy	Narrative review	3198	nd	0.1%	Cyclosporine 0.1% (Papilock mini® and Verkazia®) was effective in controlling VKC signs and symptoms	-
Yücel and Ulus [115]	2019	Turkey	Prospective observational study	30	12.9	0.05%	Topical CsA 0.05% (Restasis®) was effective in reducing VKC signs and symptoms (*p* < 0.001)	Foreign body sensation after instillation (3.3%)
Patil and Mehta [162]	2020	India	Case series	11	13.7	0.1%	Cyclosporine + tacrolimus 0.03% combined therapy allowed an improvement in VKC signs and symptoms (*p* < 0.001)	Mild irritation and burning sensation (27%)
Heikal et al. [127]	2022	Egypt	Prospective observational study	59	9.5	2%	Cyclosporine 2% was less effective than tacrolimus 0.03% in reducing individual symptoms and signs (*p* < 0.05, *p* = 0.037)	Stinging sensation in 100% of cyclosporine-treated patients
Maharana et al. [149]	2021	India	Retrospective observational study	11	13.7	0.1%	Combined use of cyclosporine and tacrolimus may lead to rapid resolution of symptoms and reduced recurrence rate in cases with severe VKC (*p* < 0.001)	Mild irritation and burning in 27% of patients with topical CsA alone and in 36% of patients with combined therapy
Bourcier et al. [130]	2022	France	Prospective observational study	46	8.8	0.1%, 2%	An improvement in symptomatic and clinical scores was observed, regardless of cyclosporine posology. There was no difference in progression between the two concentrations	Burning sensation (40%)
Salami et al. [152]	2022	Italy	Retrospective observational study	29	N/A	0.1%	CsA-CE was effective in reducing signs and symptoms in daily clinical practice. 55% of treated patients required the additional use of a 3-day course of topical dexamethasone with 1.13 ± 0.81 mean courses/month	-
Giannaccare et al. [133]	2023	Italy	Prospective observational study	25	8.4	0.1%	Symptomatic and clinical scores decreased significantly after treatment (*p* < 0.0001). Five patients (20%) required at least one course of rescue medication (mean of 3.4 ± 4.8 courses/year)	Burning sensation (8%)

*N/A* non-available information

In 2017, Thong [25] reviewed cyclosporine 0.05%, 0.1%, and 1% eye drop administration in a large cohort of VKC children, observing the efficacy of these preparations in reducing VKC signs and symptoms. Cyclosporine 0.05% was tested also in 2016 by Yücel and colleagues [115] on 20 children and adolescents with VKC, obtaining the same results. No adverse effects were reported.

Nebbioso et al. [103] focused their attention on cyclosporine 0.1% ophthalmic solution (Papilock mini® and Verkazia®). They found in the literature a cohort of 3198 patients in which the treatment with cyclosporine 0.1% eye drops administered 2–4 times a day for 4–6 months was effective in controlling VKC severe manifestations.

In 2019, Leonardi and colleagues [177] conducted a randomized clinical trial (the “Vektis Study”) that aimed to assess the efficacy and safety of cyclosporine 0.1% cationic emulsion treatment compared to a placebo in severe VKC. Patients were randomized into three groups: one group received cyclosporine eye drops 4 times a day (high-dose group), another group cyclosporine eye drops 2 times a day (low-dose group), and the third group a placebo. Patients treated with cyclosporine 4 times/day or 2 times/day showed a higher improvement in VKC signs and symptoms compared to the placebo group (*p* = 0.007 and 0.010) and had lower usage of rescue steroid eye drops (*p* = 0.010 and 0.055, respectively). Most treatment-emergent adverse events were mild or moderate in severity and consisted especially of local burning during the instillation. This finding was described also by Bremond-Gignac et al. [178], who confirmed Leonardi et al.’s conclusions also in the 8-month follow-up. The commonest adverse effects were instillation pain and pruritus.

Westland et al. in 2018 [161] described an intense regimen of 0.05% cyclosporine for vernal shield ulcers. In their case series, all three children treated with cyclosporine eight times a day showed quick resolution of the shield ulcers and complete re-epithelialization.

In 2020, Modugno et al. [126] observed that a course of cyclosporine therapy, acting at the level of epithelium, sub-basal nerve plexus, and stroma, performed progressive corneal microstructural changes, helping to restore the normal corneal microstructure (*p* < 0.001).

Borrego-Sanz et al. [167] described the case of a 10-year-old boy to whom cyclosporine was administered orally for months. In that report, daily oral cyclosporine therapy allowed the re-epithelialization of vernal shield ulcer and permitted the tapering of steroid eye drops.

Although very effective in controlling VKC signs and symptoms, 8–15% of children do not show the expected improvement with therapy. In these patients, tacrolimus eye drops may be a useful alternative [1].

Tacrolimus is an alternative therapy to cyclosporine in controlling signs and symptoms of the disease (Table 22).

**Table 22:** Tacrolimus formulations in VKC from January 2016 to June 2023

Author	Year	Country	Study design	No. of patients	Median age	Concentration	Results	Adverse effects
Al-Amri et al. [112]	2016	Saudi Arabia	Prospective observational study	20	23.1	0.1%	Significant improvement in VKC signs and symptoms (*p* < 0.001) after 6 weeks	Burning sensation
Barot et al. [113]	2016	India	Prospective observational study	36	9.3	0.1%	Important reduction of signs and symptoms (*p* < 0.0001)	Transient burning sensation (36%)
Chatterjee and Agrawal [114]	2016	India	Prospective observational study	23	14.7	0.03%	Significant improvement in signs and symptoms (*p* < 0.0001) and visual acuity (*p* = 0.05) after 12 weeks	Stinging sensation (100%)
Shoji et al. [124]	2016	Saudi Arabia	Retrospective observational study	62	12.0	0.01%	Tacrolimus permitted significant improvement in VKC symptoms and signs (*p* < 0.001)	Transient burning sensation (5%), bacterial conjunctivitis (3%)
Al-Amri et al. [117]	2017	Saudi Arabia	Prospective observational study	20	16.9	0.003%	Significant improvement in VKC symptoms and signs (*p* < 0.001) after 6 weeks	None
Liendo et al. [119]	2017	Brazil	Prospective observational study	25	12	0.03%	Important reduction in VKC signs and symptoms (*p* < 0.001)	Irritation and burning sensation (12%), ocular herpetic infection (3%)
Thong [25]	2017	Singapore	Narrative review	1800	15.7	0.005%, 0.03%, 0.1%	Tacrolimus ophthalmic solution reduced VKC signs and symptoms	-
Wan et al. [120]	2018	China	Prospective observational study	17	N/A	0.1%	Significant reductions in VKC signs and symptoms (*p* < 0.001) after 1 week	Burning sensation (29%)
González-Medina et al. [136]	2018	Spain	Retrospective observational study	17	12	0.03%	Tacrolimus permitted to stop antihistamine therapy in 8 patients (*p* < 0.05)	Burning sensation (6%)
Erdinest et al. [102]	2019	Israel	Narrative review	1121	N/A	0.003%—0.1%	The majority of patients treated with tacrolimus eye drops showed clinical improvement	Burning sensation
Fiorentini and Khurram [122]	2019	United Arab Emirates	Prospective observational study	10	N/A	0.03%	Significant reduction in signs and symptoms after 4 weeks	None
Müller et al. [140]	2019	Brazil	Retrospective observational study	21	12.0	0.03%	Tacrolimus achieved disease control without the use of steroids in 10 (47.6%) patients	Transient burning sensation, photophobia, and tearing (9%)
Liu et al. [141]	2019	Taiwan	Retrospective observational study	10	10.5	0.1%	Tacrolimus reduced conjunctival and corneal reaction (*p* = 0.0003 and 0.0002) and enabled discontinuation of steroid therapy in 6 patients (*p* < 0.05)	Burning sensation
Samyukta et al. [123]	2019	India	Prospective observational study	30	8.2	0.03%	Significant improvement in VKC signs and symptoms (*p* < 0.001) and in visual acuity (*p* = 0.04) after 8 months	Transient stinging sensation
Shoji et al. [124]	2019	Japan	Prospective observational study	1821	19.3	0.1%	Important reduction of signs and symptoms (*p* < 0.0001)	Transient burning sensation (4.1%)
Maharana et al. [149]	2021	India	Case series	11	13.7	0.03%	Cyclosporine 0.1% + tacrolimus combined therapy allowed an improvement in VKC signs and symptoms (*p* < 0.001)	Mild irritation and burning sensation in patients with combined therapy (36%)
Caputo et al. [144]	2021	Italy	Retrospective observational study	431	8.5	0.1%	All the clinical signs significantly improved during the whole follow-up in both the perennial and seasonal forms (*p* < 0.001)	Transient burning sensation
Chen et al. [180]	2021	China	RCT	76	9.9	0.1%	The combination of 0.05% azelastine and 0.1% tacrolimus eye drops leads to faster and greater improvements in clinical signs and symptoms (*p* = 0.0085)	Transient burning sensation (18.2%)
Gupta et al. [147]	2021	India	Retrospective observational study	50	8.3	0.03%	IFN α-2b was more effective than tacrolimus, had fewer side effects in the long term, and was better tolerated as compared to tacrolimus	Stinging sensation (12%) and burning sensation (36%) in the tacrolimus group
Heikal et al. [127]	2022	Egypt	Prospective observational study	59	9.5	0.03%	Tacrolimus 0.03% was more effective than cyclosporine 2% in reducing individual symptoms and signs (*p* < 0.05, *p* = 0.037)	-
Hirota et al. [148]	2021	Japan	Retrospective observational study	17	20.0	N/A	Two years of treatment with topical tacrolimus ophthalmic suspension was an effective method for inducing and maintaining the stable stages of VKC and AKC (*p* < 0.0001)	Bacterial conjunctivitis (5%), external hordeolum (3%)
Yazu et al. [150]	2021	Japan	Retrospective observational study	5	15.7	0.1%	Topical tacrolimus may provide effective and long-term improvement in clinical signs of severe AKC and VKC cases that are refractory to standard conventional treatment (*p* < 0.001)	Burning sensation (100%), elevated IOP (33%), bacterial keratitis (8%)
Saha et al. [157]	2023	India	Retrospective observational study	36	7.7	0.03%, 0.1%	Both strengths of tacrolimus (0.03% and 0.1%) are effective in recalcitrant VKC. Papillae respond better with higher strength (0.1%) but are associated with more significant side effects	Burning sensation (16%) with tacrolimus 0.1%

*N/A* non-available information

It acts on ocular inflammation [190]:

- blocking IL-2 production
- stopping the secretions of IL-3 and IL-4
- reducing mast cell degranulation

In 2017, Thong [25] reviewed the use of tacrolimus ophthalmic solution in literature in a large cohort of patients, observing its efficacy in reducing VKC signs and symptoms at various concentrations (0.005%, 0.03%, 0.1%).

Erdinest and colleagues [102] found in the literature 1121 patients treated with tacrolimus ophthalmic solutions (with a concentration variable from 0.003 to 0.1%): the larger number of patients showed clinical improvement after the treatment.

In 2016, Al-Amri et al. [112] tried a 6-week tacrolimus 0.1% therapy on 20 adult patients with VKC, concluding that the treatment allowed a significant improvement in VKC symptoms (*p* < 0.001) and signs (*p* < 0.001).

The same year, Barot and colleagues [113] also experimented with the administration of tacrolimus 0.1% ointment in VKC. That study observed an important improvement in disease control (*p* < 0.0001). About 36% of patients complained of a transient burning sensation during the treatment.

In 2018, Wan et al. [120] observed a significant improvement in signs and symptoms (*p* < 0.001) after 1 week of tacrolimus 0.1% therapy.

Tacrolimus 0.1% concentration was analyzed also in 2019 by Liu and colleagues [141]. They administered tacrolimus to ten children, observing an important reduction in conjunctival and corneal reaction (*p* = 0.0003 and 0.0002) and its consequent potential as a steroid-sparing agent. In fact, in 6 out of 10 patients, tacrolimus treatment enabled the discontinuation of steroid therapy (*p* < 0.05).

In 2017, Al-Amri and his team [117] administered a less concentrated tacrolimus 0.003% therapy for 6 weeks to 20 adolescents affected by a severe and resistant form of VKC. In all patients, the prescribed therapy permitted a significant improvement in VKC symptoms (*p* < 0.001) and signs (*p* < 0.001), with no important side effects.

In 2019, Samyukta et al. [123] tried an 8-month treatment with a more concentrated tacrolimus ophthalmic solution (0.3%) in 30 children with VKC, observing an important decrease in VKC’s signs and symptoms (*p* < 0.001) and an improvement in visual acuity (*p* = 0.04).

In 2019, Shoji et al. [124] wanted to evaluate if the efficacy of tacrolimus ophthalmic solution was different in patients with concomitant atopic dermatitis or not. To do so, they enrolled a cohort of 1821 adolescents and young adults affected by the chronic allergic conjunctival disease (AKC or VKC) with and without atopic dermatitis. Tacrolimus therapy showed its efficacy in reducing ocular signs and symptoms in both groups (*p* < 0.0001). The concomitant use of topical steroids significantly increased the likelihood of remission (*p* < 0.0001).

A 0.03% tacrolimus ophthalmic solution was tested in 2016 by Chatterjee and Agrawal [114] with the administration of that drug in 23 adolescents with VKC. In his study, symptoms and signs were significantly reduced at 4 and 12 weeks (*p* < 0.0001). Furthermore, visual acuity showed an improvement after 12 weeks of treatment (*p* = 0.05).

The same results were observed also in 2019 when Fiorentini and Khurram [122] administered tacrolimus 0.03% ointment in 10 Arabian children with VKC. After a 4-week course of therapy, all subjects showed an improvement in their symptomatology without any adverse effects. Müller and colleagues [140] achieved similar results in the same year. In fact, in their VKC patients, topical tacrolimus 0.03% ointment achieved disease control. Furthermore, in 47.6% of patients, steroid treatment could be interrupted.

González-Medina et al. [136] tested 17 adolescents with VKC 0.03% tacrolimus eye ointment. The therapy permitted the cessation of antihistamine therapy in 8 patients (*p* < 0.05). The number of flare-ups per year was not reduced, but the duration and the severity of each exacerbation were reduced.

In the literature, several studies with a minimal concentration of tacrolimus are reported, such as the 0.01% tested by Shoughy et al. in Saudi Arabia [135]. In his study, 62 children with VKC have been treated with tacrolimus 0.01% ophthalmic solution, with an important improvement in VKC signs and symptoms (*p* < 0.001).

In 2017, Zanjani et al. [176] conducted an RCT that aimed to compare the efficacy of tacrolimus 0.005% versus interferon alpha-2b (IFN alpha-2b) eye drops in the treatment of VKC. Both patients treated with tacrolimus and patients treated with IFN alpha2b showed an improvement in VKC signs and symptoms after 3 years (*p* < 0.0001 for both groups), without significant statistical difference between the two groups (*p* > 0.05). No major ocular complications or systemic side effects related to tacrolimus and IFN alpha-2b were noted.

The authors concluded that both 0.005% tacrolimus and IFN alpha-2b might be promising and effective treatments for resistant VKC.

The same year, Gayger Müller and colleagues [175] evaluated the efficacy of tacrolimus versus sodium cromoglycate monotherapy in VKC. With their RCT, they treated eight patients with tacrolimus 0.03% eye drops and eight patients with sodium cromoglycate. Tacrolimus was more effective than sodium cromoglycate in controlling VKC signs and symptoms (*p* = 0.001 and 0.015).

In 2021, Maharana et al. [149] performed a combined local therapy with cyclosporine 0.1% and tacrolimus 0.03% in 11 VKC patients, observing that the combination was very helpful in improving VKC signs and symptoms (*p* < 0.001).

In 2022, Heikal et al. [127] demonstrated that tacrolimus 0.03% permitted a reduction in individual symptoms and signs better than cyclosporine 2% eye drops.

In 2021, the independent study of Caputo et al. [144], Hirota et al. [148], and Yazu et al. [150] demonstrated retrospectively how long-term use of topical tacrolimus is a safe option for refractory VKC.

In fact, like cyclosporine, tacrolimus is generally well tolerated. The only side effect reported is burning at the drops’ instillation. Being an immunosuppressive agent, it could also increase the risk of ocular infections.

If administered orally, it is toxic to the kidney and the neural system. It could also provoke hypertension, diabetes, infections, tumors, and gastrointestinal disorders [190]. If administered topically in the eye, the systemic absorption of tacrolimus is nearly zero; thus, systemic adverse effects have never been reported in the literature [31].

### Monoclonal Antibodies

Omalizumab is an anti-IgE monoclonal antibody. It was created to treat allergic asthma, but, in recent years, its use has been extended also to the treatment of other allergic conditions, like atopic dermatitis, chronic urticaria, allergic rhinitis, allergic bronchopulmonary aspergillosis, and food allergy [191]. In the last years, omalizumab has been tested also in the treatment of recalcitrant VKC, with good results (Table 23).

**Table 23:** Omalizumab treatment in VKC from January 2016 to June 2023

Author	Year	Country	Study design	No. of patients	Median age	Results	Adverse effects
Heffler et al. [158]	2016	Italy	Case series	2	N/A	With the monthly administration of omalizumab, both patients had an improvement in VKC symptoms, physical examination, and conjunctival cytologic findings. Omalizumab was an effective treatment in patients with VKC without concomitant asthma	None
Doan et al. [16]	2017	France	Case series	4	9.2	Omalizumab was administered every 2 weeks for 8 weeks. 3 of 4 patients responded to the treatment, but the response was incomplete	None
Occasi et al. [159]	2017	Italy	Case series	4	8.5	After a 6-month omalizumab therapy, all children experienced an improvement of ocular symptoms and signs. No relapse was observed after treatment suspension	None
Callet et al. [160]	2018	France	Case series	2	8.0	Monthly omalizumab therapy permitted VKC and asthma control in both patients	None
Santamaría and Sánchez [165]	2018	Colombia	Case report	1	15	The bi-weekly use of omalizumab has proved effective in the treatment of VKC. However, upon discontinuation of the drug, the symptoms resumed	None
Simpson and Lee [166]	2018	Canada	Case report	1	54	A single injection of omalizumab resolved VKC signs and symptoms in the adult patient	None

*N/A* non-available information

The only adverse effects reported in the literature are pain at the injection site, headache, pharyngitis, upper respiratory tract symptoms, and sinusitis [191].

Doan et al. [16] performed a literature review evaluating the efficacy of omalizumab therapy in severe refractory VKC. Omalizumab, allowing the reduction of signs and symptoms, appeared to be a potent treatment for refractory forms of VKC. The strongest evidence was provided by Doan and colleagues, who administered omalizumab to four children aged 7–13 years. Three out of 4 patients responded to the treatment, but the response was incomplete.

Other studies describing the use of omalizumab in VKC patients were conducted by Heffler et al. [158] (2 patients, both showed an improvement in VKC symptoms, physical examination, and conjunctival cytologic findings), Occasi et al. [159] (4 children aged 6–11 years, all of whom responded to omalizumab therapy without any side effects), Callet et al. [160] (2 children aged 7–9 years, all of them had an improvement in VKC and asthma control), Santamaría and Sánchez [165] (1, 15-year-old patient who had improvement of VKC symptoms once omalizumab was administered; however, upon discontinuation of the drug, the symptoms relapsed), and Simpson and Lee [166] (1 adult with VKC, in which a single dose of omalizumab appeared to resolve all the signs and symptoms of VKC).

In literature, omalizumab has not been the only monoclonal antibody used in VKC, albeit the most widely studied.

In 2022, Tsui et al. [192] administered dupilumab, a human monoclonal antibody against interleukin (IL)-4 receptor alpha, to three children affected by refractory VKC (aged 7–14 years), obtaining total control of VKC signs and symptoms within 1 month of treatment. Dupilumab treatment also resulted in resolution of shield ulcer, corneal re-epithelialization, and complete resolution of giant papillae on the upper tarsal conjunctiva in all patients. However, it should be remembered that in literature treatment with dupilumab is associated with the development of dry eye and conjunctivitis as an adverse reaction [193]. The appearance of side effects in patients treated with dupilumab for atopic dermatitis, already extensively described in the literature [194, 195], has made it possible to demonstrate the efficacy of upadacitinib, a JAK2 inhibitor, in a case of atopic dermatitis severe and AKC [196]. To our knowledge, upadacitinib has not yet been tested in patients with VKC.

In 2022, Anesi et al. [197] tried the administration of lirentelimab, a monoclonal antibody against sialic acid-binding immunoglobulin-like lectin (Siglec)-8, in a 25-year-old man with VKC, asthma, and allergic rhinitis, founding that lirentelimab was well tolerated, improved VKC symptoms and concomitant allergic symptoms, and reduced inflammatory mediators in patient tears.

Other monoclonal antibodies, such as mepolizumab, reslizumab, and benralizumab, are under investigation for their efficacy in eosinophilic asthma [106, 193] and may also be useful in other allergic diseases and VKC.

Clinical trials are needed to investigate their potential therapeutic benefits in other types of eosinophil-mediated conditions, such as VKC.

### Other Drugs

The last year, a large cohort study by Xu and Cai [125] aimed to evaluate the therapeutic effects and safety of houttuynia eye drops combined with olopatadine hydrochloride in VKC patients. They observed that children treated with the association of houttuynia and olopatadine eye drops showed a rapid reduction in VKC symptoms (*p* < 0.05), without adverse effects.

### Surgical Treatment

Surgical treatment used in the VKC is summarized in Table 24.

**Table 24:** Surgical treatment for VKC from January 2016 to June 2023

Author	Year	Country	Study design	No. of patients	Median age	Results	Adverse effects
Das et al. [163]	2016	India	Case report	1	22	Amniotic membrane transplantation, followed by cataract surgery and optical prosthetics, was effective in the treatment of VKC complications	None
Abozaid [116]	2017	Egypt	Prospective observational study	11	13.6	Femtosecond laser-assisted Keraring implantation followed by transepithelial accelerated corneal collagen cross-linking (CXL) for the treatment of keratoconus in children with VKC permitted an improvement in visual acuity, keratometry values, and refraction (*p* < 0.001)	None
Iyer et al. [137]	2018	India	Retrospective observational study	6	10.6	After mucous membrane grafting (MMG) or refractory giant papillae, reactivation of allergic activity was noted in all the eyes, but with no recurrence of shield ulcers or diffuse punctate keratitis	None
Abozaid et al. [138]	2019	Egypt	Retrospective observational study	28	14.3	Femtosecond laser-assisted intrastromal corneal ring segments’ (ICRS) implantation followed or accompanied by transepithelial accelerated corneal collagen cross-linking (TE-ACXL) in the treatment of keratoconus permitted the achievement of better visual acuity (*p* = 0.001) and corneal measure (*p* < 0.001) than patients treated with CXL only	Acute keratitis (3.7%)
Alrobaian et al. [139]	2019	Saudi Arabia	Retrospective observational study	19	15.8	Corneal collagen cross-linking (CXL) is a safe and effective strategy to treat keratoconus in VKC patients	Progression of keratoconus (18.5%)
Hopen et al. [168]	2019	USA	Case report	1	8	To report a case of intraocular pressure (IOP) reduction after a gonioscopy-assisted transluminal trabeculotomy in a VKC children. Vision and IOP showed improvement	Small hyphema
Iqbal et al. [179]	2020	Egypt	Randomized clinical trial	38	14.3	There were significant differences in visual acuity and refractive measure between the three groups throughout the study (*p* < 0.0001) in favor of standard epithelium-off cross-linking (SCXL) followed by accelerated epithelium-off cross-linking (ACXL). SCXL protocol was superior to ACXL and transepithelial epithelium-on cross‐linking (TCXL), with an overall success rate of SCXL being 100% during 2 years of follow‐up	Photophobia (24.7%), delay in epithelial healing (7.7%), persistent epithelial defect (0.7%), corneal stromal opacity (0.3%), corneal haze (28.7%), keratoconus progression (11.0%)
Stock et al. [17]	2020	Brazil	Case series	2	5.5	In both cases, surgical debridement was curative and definitive in the 7-month follow-up period. Shield ulcer did not recur	
Elubous et al. [145]	2021	Jordan	Retrospective observational study	20	31.2	VKC is a statistically significant risk factor (*p* = 0.005) for penetrating keratoplasty (PKP) in patients with keratoconus	/
Feizi et al. [146]	2021	Iran	Retrospective observational study	117	25.4	There is no difference in outcomes between penetrating keratoplasty (PK) and deep anterior lamellar keratoplasty (DALK) for keratoconus in patients with VKC	Suture complications (58.9%), increased intraocular pressure (6.8%), graft rejection (30.5%)
Singh et al. [171]	2021	India	Case report	1	17	Amniotic membrane transplantation (AMT) along with penetrating keratoplasty (PK) is a simple and effective modality of treatment for limbal stem cell deficiency	None
Jain et al. [172]	2022	India	Case report	1	22	The patient underwent a cadaveric allo simple limbal epithelial transplantation in the right eye to restore the ocular surface. Systemic immunosuppression with oral cyclosporine was administered. The corrected visual acuity was 20/20 in both eyes. No recurrence of limbal stem cell deficiency was observed	None
Kate et al. [173]	2022	India	Case report	1	32	The patient underwent excision of the conjunctival keratinization in both eyes. The resultant bare areas were covered with conjunctival autografts	None
Patil and Mehta [162]	2022	Singapore	Case series	4	9.8	Surgical excision of giant papillae in combination with mitomycin C and amniotic membrane transplantation in refractory VKC is a good treatment option with better clinical outcomes over a longer follow-up	None
Senthil et al. [153]	2022	India	Retrospective observational study	82	15.8	The surgical success for trabeculectomy, trabeculectomy with mitomycin C, and combined trabeculectomy with cataract extraction is similar at 5 years. Chronic VKC and long‑term steroid use are associated with surgical failure	None
Arora et al. [154]	2023	India	Retrospective observational study	3	17.0	One year after repeat deep anterior lamellar keratoplasty (DALK) in patients of previous failed DALK, best-corrected visual acuity improved from 20/120 to 20/30 in all except one patient	None

*N/A* non-available information

Stock et al. [17] reviewed surgical debridement of VKC shield ulcers in the literature. They found only four studies on VKC patients, and in all of them, the surgical debridement proved extremely effective in the treatment of shield ulcers. The procedure was followed by a rapid corneal re-epithelialization, and no adverse effects were described. They described also their experience with two children treated with surgical debridement of the ulcer, in which the surgical treatment was curative and definitive in the 7-month follow-up period.

In 2017, Abozaid [116] tried to assess the safety and efficacy of femtosecond laser-assisted Keraring implantation followed by transepithelial accelerated corneal collagen cross-linking (CXL) for the treatment of keratoconus in children with VKC. In their observational study, all the eyes treated showed an improvement in visual acuity, keratometry values, and refraction (*p* < 0.001). No intraoperative complications were reported.

Also, Alrobaian and colleagues [139] performed a retrospective study to determine the safety and efficacy of corneal collagen cross-linking (CXL) in patients with keratoconus and VKC. However, in the 19 patients treated, they did not observe a significant difference between the baseline and last follow-up of visual acuity (*p* = 0.99) and keratometry values (*p* = 0.093). Furthermore, 5 of 27 eyes with VKC exhibited progression of keratoconus (18.5%).

In 2019, Abozaid et al. [138] conducted a retrospective observational study of 28 adolescents with VKC to evaluate femtosecond laser-assisted intrastromal corneal ring segment (ICRS) implantation followed or accompanied by transepithelial accelerated corneal collagen cross-linking (TE-ACXL) as a treatment of keratoconus in VKC. In that study, they observed better visual acuity (*p* = 0.001) and corneal measure (*p* < 0.001) in patients treated with Keraring + CXL with respect to patients treated with CXL only.

In 2020, Iqbal et al. [179] performed a controlled trial to compare standard epithelium-off cross-linking (SCXL) versus accelerated epithelium-off cross-linking (ACXL) and transepithelial epithelium-on cross-linking (TCXL) in the treatment of keratoconus in children. One hundred thirty-six patients with keratoconus (of whom 38 had also VKC) were assigned to SCXL, ACXL, or TCXL surgical treatment. The author observed significant differences in visual acuity and refractive measure between the three groups throughout the study (*p* < 0.0001) in favor of SCXL followed by ACXL. SCXL protocol was superior to ACXL and TCXL, with an overall success rate of SCXL of 100% during 2 years of follow-up.

In 2018, Iyer et al. [137] published a retrospective observational study aimed to evaluate the outcomes of mucous membrane grafting (MMG) for refractory giant papillae in VKC. Six children were treated with MMG. After the surgery, reactivation of the allergic activity was noted in all the eyes, but with no recurrence of shield ulcers or diffuse punctate keratitis.

In 2019, Hopen et al. [168] reported a case of intraocular pressure (IOP) reduction after a gonioscopy-assisted transluminal trabeculectomy (GATT) in a VKC child, in which the only adverse effect was a small hyphema.

In 2016, Das et al. [163] described the case of a 22-year-old man affected by VKC who underwent amniotic membrane transplantation, followed by cataract surgery and optical prosthetics for the treatment of VKC complications, with overall good results.

In 2022, Senthil et al. [153] compared in a retrospective observational study the surgical success rate for trabeculectomy, trabeculectomy with mitomycin C, and combined trabeculectomy with cataract extraction for glaucoma’s treatment, founding it similar at 5-year follow-up. All the three surgical techniques proved to be effective, but the surgical result is inversely proportional to the age of the child, the duration of VKC, the duration of steroid therapy, and mixed type of steroid use.

In the literature, it is a common idea that VKC should be treated “step-by-step.” Most of the authors (Fauquert et al. [27], Takamura et al. [24], Gokhale et al. [23], Berger et al. [19], Sacchetti et al. [26], Esposito et al. [101], AlHarkan et al. [104], Maitra et al. [121], Kraus [18]) agreed to reserve cyclosporine and tacrolimus eye drops and surgical measures at the severest form of VKC, while mild and moderate forms should be treated with antihistamines and cycles of steroid eye drops. Also, the systematic review of Singhal et al. [98] remarked that surgical therapy (like corneal ulcer debridement or resection of giant papillae) should be performed only in severe giant papillary hypertrophy or refractory shield ulcer, while the majority of VKC patients could be managed with medication alone.

## Conclusions

VKC is a disease of the anterior chamber of the eye with an unclear etiology. The diagnosis is clinical, as no safe markers of the disease and its severity have yet been identified. Similarly, no markers have been established that can be used for follow-up.

It would be desirable to draw up a score based on standardized and shared parameters of objective signs, subjective symptoms, and possible presence of complications. The score should be corrected based on the geographical reality and the season in which it is detected, to make the data collected comparable and evaluate the effectiveness of the therapy at different latitudes.

In the literature, the graduality of the therapy is described, but without clear objective parameters on which to base its modification. In some cases, the risk is beginning immunomodulatory therapy when the lesions are already too advanced.

The use of biotechnological drugs should also be studied, in the absence of an accurate study of the inflammatory cytokines present in the eye and in the absence of methods for the determination of these cytokines at the tear level that can be used in clinical routine.

## Data Availability

Data available on reasonable request.
